# Visualizing transfer of microbial biomolecules by outer membrane vesicles in microbe‐host‐communication in vivo

**DOI:** 10.1002/jev2.12159

**Published:** 2021-10-19

**Authors:** Miriam Bittel, Patrick Reichert, Ilann Sarfati, Anja Dressel, Stefanie Leikam, Stefan Uderhardt, Iris Stolzer, Tuan Anh Phu, Martin Ng, Ngan K. Vu, Stefan Tenzer, Ute Distler, Stefan Wirtz, Veit Rothhammer, Markus F. Neurath, Robert L. Raffai, Claudia Günther, Stefan Momma

**Affiliations:** ^1^ Department of Medicine 1 Friedrich‐Alexander‐University Erlangen‐Nürnberg Erlangen Germany; ^2^ Deutsches Zentrum Immuntherapie Friedrich‐Alexander University Erlangen‐Nürnberg Erlangen Germany; ^3^ Department of Internal Medicine 3 University Hospital Erlangen and Friedrich‐Alexander‐University Erlangen‐Nürnberg (FAU) Erlangen Germany; ^4^ Exploratory Research Unit Optical Imaging Centre Erlangen Friedrich‐Alexander‐University Erlangen‐Nürnberg (FAU) Erlangen Germany; ^5^ Northern California Institute for Research and Education San Francisco California USA; ^6^ Institute of Immunology University Medical Centre of the Johannes‐Gutenberg University Mainz Mainz Germany; ^7^ Research Centre for Immunotherapy (FZI) University Medical Center of the Johannes‐Gutenberg University Mainz Mainz Germany; ^8^ Neurology Department (Experimental Glia Biology) University Hospital Erlangen and Friedrich‐Alexander‐University Erlangen‐Nürnberg (FAU) Erlangen Germany; ^9^ Department of Surgery Division of Vascular and Endovascular Surgery University of California San Francisco San Francisco California USA; ^10^ Department of Veterans Affairs Surgical Service (112G) San Francisco VA Medical Centre San Francisco California USA; ^11^ Institute of Neurology (Edinger Institute) Goethe University Frankfurt am Main Germany

**Keywords:** Cre‐Loxp, extracellular vesicles, inflammation, intestinal stem cells, microbiome, outer membrane vesicles

## Abstract

The intestinal microbiota influences mammalian host physiology in health and disease locally in the gut but also in organs devoid of direct contact with bacteria such as the liver and brain. Extracellular vesicles (EVs) or outer membrane vesicles (OMVs) released by microbes are increasingly recognized for their potential role as biological shuttle systems for inter‐kingdom communication. However, physiologically relevant evidence for the transfer of functional biomolecules from the intestinal microbiota to individual host cells by OMVs in vivo is scarce. By introducing *Escherichia coli* engineered to express Cre‐recombinase (*E. coli* ^Cre^) into mice with a *Rosa26.tdTomato*‐reporter background, we leveraged the Cre‐LoxP system to report the transfer of bacterial OMVs to recipient cells in vivo. Colonizing the intestine of these mice with *E. coli* ^Cre^, resulted in Cre‐recombinase induced fluorescent reporter gene‐expression in cells along the intestinal epithelium, including intestinal stem cells as well as mucosal immune cells such as macrophages. Furthermore, even far beyond the gut, bacterial‐derived Cre induced extended marker gene expression in a wide range of host tissues, including the heart, liver, kidney, spleen, and brain. Together, our findings provide a method and proof of principle that OMVs can serve as a biological shuttle system for the horizontal transfer of functional biomolecules between bacteria and mammalian host cells.

## 1 INTRODUCTION

The gut microbiota is considered an ‘essential organ’ that contains approximately 150 times more genes than are found in the entire human genome (Ursell et al., 2014). This microbial ecosystem critically influences its host and plays a crucial role in basic biological processes such as the development and maturation of immune cells, regulation of metabolic processes, and intestinal homeostasis by influencing epithelial turnover and differentiation (Belkaid & Hand, 2014). Thus, it is not surprising that alterations in gut microbial composition have been shown to contribute pathogenic factors in a variety of immune‐ and metabolically driven diseases such as autoimmune‐ and neoplastic diseases (Levy et al., 2017). Recent studies have also linked alterations in microbial composition to multiple pathologies in organs devoid of direct contact with the intestinal microbiota, such as the liver, joints and the brain (Albillos et al., 2020; Ma et al., 2019; Rastelli et al., 2018). However, there is a growing awareness of the need to improve our understanding of causality regarding the characterization of molecular mechanisms by which bacteria elicit their beneficial or pathogenic effects on individual host cells (Koh & Bäckhed, 2020). So far, research on bacterial‐host communication regarding bioactive molecules, such as enzymes and toxins, has been mainly focused on direct transmission of biomolecules from bacteria to mammalian host cells via different secretion systems during cell surface‐attachment or intracellular‐vacuolization (Green & Mecsas, 2016). However, several important functions of the gut microbiota for host physiology, for example, education of the immune system, are difficult to explain by direct host‐microbe interaction since microbes normally remain in the gut lumen.

Accordingly, increasing evidence is pointing towards an alternative inter‐kingdom communication system by which functional molecules can be transmitted from bacteria to their host without direct cell‐cell contact via bacterial‐derived extracellular vesicles (BEVs) (Tsatsaronis et al., 2018). In general, EVs are broadly defined as small membrane‐bound vesicles released by virtually all cells and living organisms including bacteria, archaea, fungi and protozoa (Coelho & Casadevall, 2019; Deatherage & Cookson, 2012). BEVs released by gram‐negative bacteria are generally referred to as outer membrane vesicles (OMVs).

Both pathogenic and commensal gram‐negative bacteria produce such OMVs as part of their normal growth as well as a bacterial stress response to promote nutrient acquisition, biofilm development and pathogenesis (Kaparakis‐Liaskos & Ferrero, 2015; Kulp & Kuehn, 2010; Li et al., 2020; Schwechheimer & Kuehn, 2015). These small vesicles are enveloped with lipid‐bilayer membranes released from the outer membrane of bacteria with an average size ranging from approximately 20 to 250 nm (Kulp & Kuehn, 2010). Previous OMIC analyses have uncovered that OMVs contain much of the biological material found within the parental bacterium including nucleic acids, enzymes and toxins, polysaccharides and peptidoglycan and thus represent non‐replicative mimics (Kadurugamuwa & Beveridge, 1995; Kulp & Kuehn, 2010; Lee et al., 2016; Renelli et al., 2004; Whitchurch et al., 2002). Importantly, OMVs can disseminate far away from their parental bacterium and thus have been proposed to serve as a mechanism of intra‐kingdom communication enabling the transfer of embodied bioactive molecules (Blenkiron et al., 2016; Koeppen et al., 2016). Moreover, there is increasing in vitro evidence pointing towards an important role of this shuttle system for host‐microbe communication (Tsatsaronis et al., 2018). Accordingly, OMVs produced by commensals have been shown to be essential for the maturation of the immune system, while pathogens release OMVs that facilitate inflammation and infection in the host (Durant et al., 2020; Hickey et al., 2015; Irving et al., 2014; Kaparakis‐Liaskos & Ferrero, 2015; Shen et al., 2012; Zingl et al., 2020). Indeed, OMVs have been identified in a wide range of human biofluids and tissues (Craven et al., 1980; DeVoe & Gilchrist, 1975; Fiocca et al., 1999; Keenan et al., 2000; Stephens et al., 1982; Vidakovics et al., 2010), which strengthened the idea that OMVs might contribute to disease pathogenesis and progression.

Although multiple lines of evidence point towards an essential role of OMVs for host‐microbe communication, suitable in vivo models for proof‐of‐concept studies addressing the horizontal transfer of functional bacterial molecules by BEVs to mammalian host cells, as well as a fundamental understanding of the distribution and ultimate impact of these EVs on host physiology and pathophysiology are lacking (Stentz et al., 2018). Results of our study provide, to the best of our knowledge, the first direct evidence of horizontal transfer of functional biomaterial from gut bacteria to individual host cells in diverse mammalian tissues and organs via OMVs.

## 2 RESULTS

### 2.1 OMVs as shuttle system for functional Cre protein and RNA to epithelial cells

**FIGURE 1:**
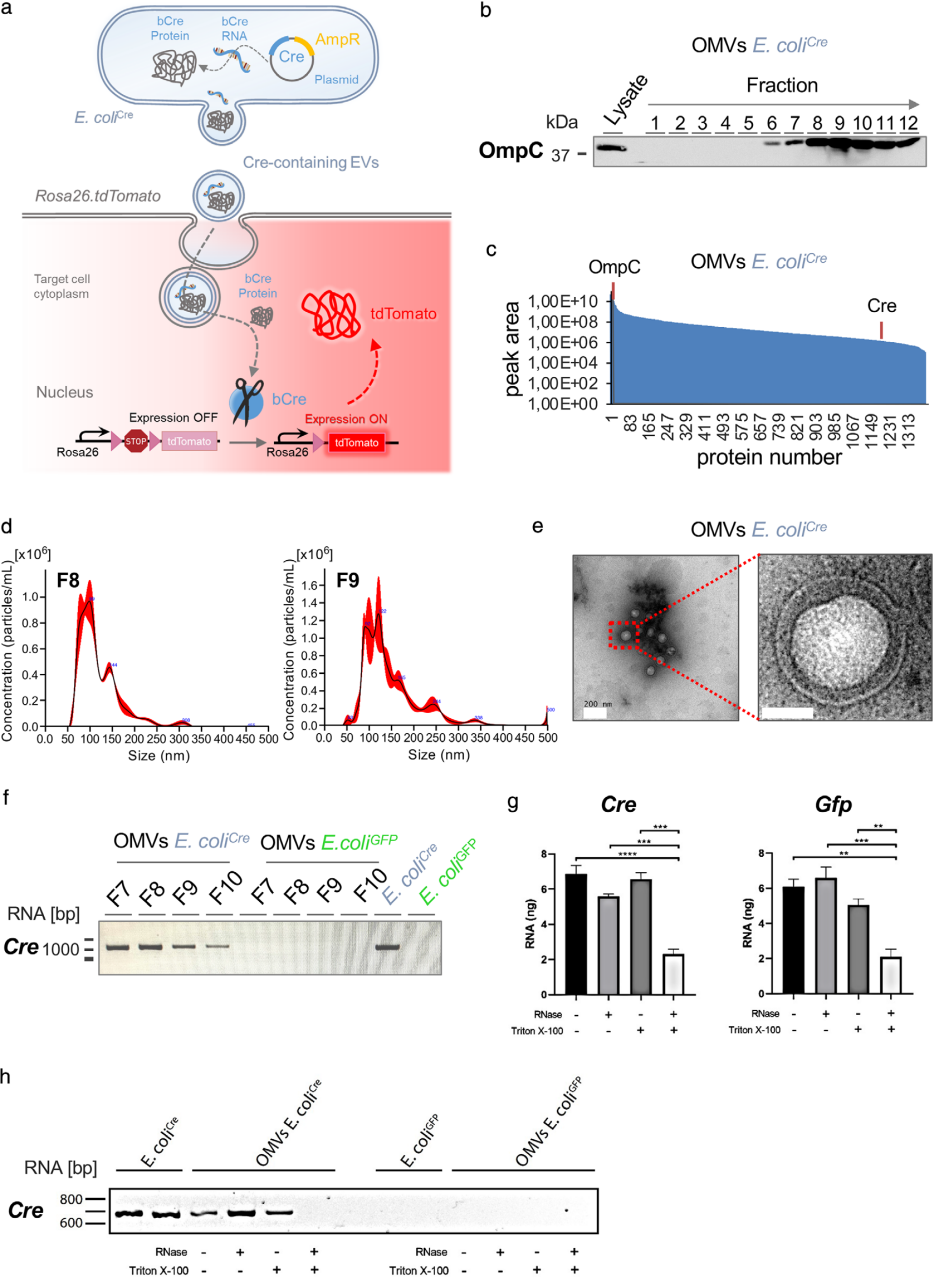
Characterization of OMVs. (a) Graphical illustration of bacterial OMVs transferring their molecular cargo to mammalian host cells. *E. coli* ^Cre^‐derived OMVs containing Cre RNA or protein are taken up by host cells of *Rosa26.tdTomato* reporter mice, leading to Cre‐mediated excision of the *loxP*‐flanked STOP cassette, thereby inducing expression of robust *tdTomato* fluorescence (red). (b) Western blot analysis using 37,5 μl of each fraction derived from iodixanol density gradient purification as well as bacterial lysates (5 μg) of *E. coli* ^Cre^. The membrane was probed with an antibody against OmpC (37 kDa). (c) Dynamic range of OMV contained proteins analyzed by mass spectrometry, relative quantity of OmpC and Cre are indicated by orange lines. (d) Representative NTA analysis of fraction 8 and 9 of iodixanol density gradient purified *E. coli* ^Cre^ OMVs. (e) Transmission electron microscopic image of OMV preparation isolated from *E. coli* ^Cre^ culture supernatant showing membranous particles confirming the presence of small OMVs. Scale Bar: 200 nm and 50 nm. (f) RT‐PCR analysis of full length Cre RNA from fractions F7 to F10 of iodixanol density gradient purified OMVs and whole bacterial lysates from *E. coli* ^Cre^ and *E. coli* ^GFP^. (g) Isolated OMVs (OMVs *E. coli* ^Cre^ and OMVs *E. coli* ^GFP^) were left unchallenged or treated with RNAse or Triton X‐100 or a combination of the two. RNA concentration was measured by Quant‐iT RiboGreen RNA Assay. (h) RT‐PCR analysis of Cre RNA from whole bacterial lysates and iodixanol density gradient purified OMVs (from *E. coli* ^Cre^ and *E. coli* ^GFP^) left unchallenged or treated with RNAse, Triton X‐100 or a combination of both.

To address this emerging field in microbe‐host communication, we adapted a robust in vivo model (based on the Cre‐LoxP system), that we previously established (Ridder et al., 2014, 2015) to visualize mammalian cell‐cell communication via EVs in vivo. Utilizing this strategy, colonization of *Rosa26.tdTomato* reporter mice with a Cre‐expressing *E. coli* strain (*E. coli^Cre^*) would allow us to identify individual host cells targeted by Cre that is delivered from bacteria to host cells via OMVs (Figure 1a). In this model, transfer of functional bacterial derived Cre (protein or RNA) leads to the excision of the *loxP*‐flanked STOP cassette prior to the reporter gene tdTomato under the control of the CAG promoter, resulting in the continuous expression of the fluorescent tdTomato within the host cell (Figure 1a). To obtain a pure population of OMVs, we first sedimented them from bacterial supernatant using PEG and subsequently floated the pellet in an optiPrep iodixanol density gradient using ultracentrifugation and collected twelve fractions from the top. We then investigated these fractions for the presence of OmpC (Outer membrane porin C), an outer membrane protein that is also present on OMVs (Thoma et al., 2018). Based on the presence of OmpC as well as Cre RNA and after additional visual inspection of OMV preparations by electron microscopy, we decided to subsequently use a pool of fractions 7–9 with densities ranging between 1.08 and 1.10 g/ml for further analyses (Figure 1b). Liquid Chromatography‐Mass Spectrometry analysis confirmed that OmpC was indeed the most abundant protein present in OMVs (Figure 1c). In addition, we could also detect Cre protein in our OMV preparation, but not in the supernatant after ultracentrifugation (Figure 1c, Table S1). Nanoparticle tracking analysis (NTA) (Figure 1d) and transmission electron microscopy images (Figure 1e) of iodixanol density gradient purified OMVs (F8, F9) revealed that *E. coli* ^Cre^ OMVs display a nano‐sized lipid‐bilayered vesicular structures with a predominant size between 50 nm and 150 nm. As previous OMIC analyses revealed that OMVs contain nucleic acid as well as proteins (Schwechheimer & Kuehn, 2015; Tsatsaronis et al., 2018), in a next step we investigated, if OMVs contain *Cre* RNA in addition to Cre protein. Therefore, we performed mRNA analysis on isolated OMVs derived from *E. coli* ^Cre^ and a control *E. coli* strain (*E. coli* ^GFP^), respectively. We could detect full length bacterial *Cre*‐mRNA derived from lysed *E. coli* ^Cre^ bacteria and *E. coli* ^Cre^ OMVs from fractions F7 to F10, but not from bacterial culture supernatant after ultracentrifugation nor from equivalently processed bacteria or OMV preparations of control *E. coli* ^GFP^ (Figure 1f). To quantify the amount of total RNA and to ascertain the localization of RNA within OMVs, we performed an RNA protection assay, showing that OMV‐enclosed RNA was indeed protected from degradation by RNAses added to OMV preparations. Only further addition of a detergent to the OMV fractions resulted in the degradation of the RNA‐protecting OMV envelope leading to a measurable reduction of RNA concentration in the double‐treated OMV samples (Figure 1g). This finding could be confirmed for equivalently treated *E. coli^Cre^* OMV samples via RT‐PCR, where a signal for *Cre* RNA was lost only after simultaneous treatment with RNase and Triton X‐100 (Figure 1h). Taken together, our results provide compelling evidence for OMVs as cargo for microbial RNA and protein, and as a potential shuttle system for functional Cre delivery to mammalian host cells in this model.

### 2.2 OMVs are taken up by epithelial cells via endocytosis in vitro

**FIGURE 2:**
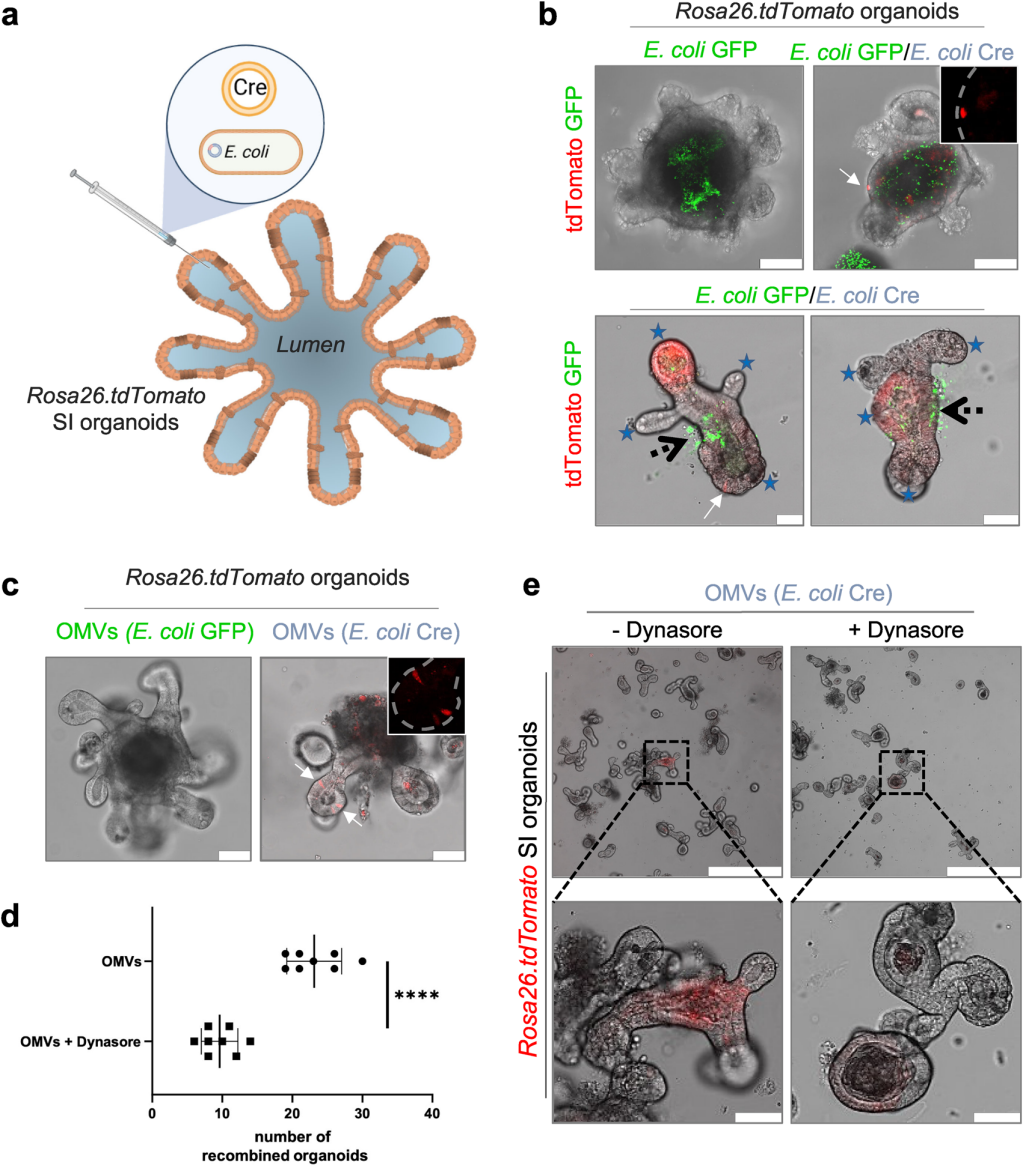
OMV uptake by intestinal epithelial cells. (a) Experimental set up of in vitro experiments. Organoids received either 4*10^4^ bacteria or 4*10^5^ OMVs via microinjection. (b) Small intestinal organoids derived from *Rosa26.tdTomato* mice were microinjected with *E. coli* ^GFP^ (left, Green) or *E. coli* ^Cre^ plus *E. coli* ^GFP^ (right, Green 1:1 dilution). Pictures were taken 8 (upper panel) or 20 (lower panel) hours post injection. Successful transfer of bacterial Cre to host cells is traceable by tdTomato‐positivity (red). Scale Bar: 50 μm. Inset: Higher magnification of tdTomato‐positive signal only (red). Experiments were repeated three times with similar results. Stars mark crypt region, black arrows label side of microinjection, white arrow marks individual tdTomato positive epithelial cell. (c) Representative confocal pictures of small intestinal organoids (*Rosa26.tdTomato*) microinjected with OMVs derived from *E. coli* ^GFP^ (left) or *E. coli* ^Cre^ (right). Confocal images were taken 8 hours after microinjection. Experiments were repeated three times with similar results. (d) Quantification and (e) representative confocal images of small intestinal organoids derived from *Rosa26.tdTomato* mice after microinjection with purified OMVs (*E. coli* ^Cre^) cultured in the absence (‐Dynasore) or presence (+Dynasore) of the dynamin GTPase inhibitor Dynasore. The data are depicted as the mean of the number of organoids that displayed tdTomato positive cells (independent of intensity). The dot plots depict the mean of one analysed culture [with 100 microinjections] and the whiskers min–max values. ****P < 0.0001. Data are derived from two independent experiments.

In order to provide functional evidence for OMVs as a shuttle system for horizontal transfer of bioactive molecules, we investigated if OMVs derived Cre was sufficient to induce reporter gene expression in intestinal epithelial cells, which represent the first line of defence within the host system. To study this inter‐kingdom communication on a cellular level, we took advantage of the intestinal organoid culture system. To this end, we generated small intestinal organoids derived from *Rosa26.tdTomato* reporter mice and introduced *E. coli* ^Cre^ together with *E. coli* ^GFP^ (to visualize the bacteria) or *E. coli* ^GFP^ alone (as a control) via microinjection into the lumen of these organoids to mimic the physiological site of interaction (Figure 2a). Six hours post microinjection we were able to detect single tdTomato‐positive cells in intestinal organoids injected with *E. coli^Cre^* but not in those injected with *E. coli* ^GFP^ (Figure 2b, upper panel). Twenty hours post injection we observed fully tdTomato positive buds (area of progenitor cells and Paneth cells) in organoids that were co‐cultured with *E. coli^Cre^* (Figure 2b, lower panel). These data implicate that OMVs are capable of transferring bioactive molecules to intestinal epithelial cells in vitro. To provide direct functional evidence that OMVs transfer functional bacterial biomolecules to mammalian host cells, we injected purified OMVs derived from *E. coli* ^Cre^ or *E. coli* ^GFP^ (as control) into *Rosa26.tdTomato* intestinal organoids. Again, expression of *tdTomato* could only be observed in organoids injected with *E. coli^Cre^* OMVs (Figure 2c) indicating that OMVs are sufficient to transfer bacterial Cre to induce *tdTomato* expression in intestinal epithelial cells. Of note, western blot analysis of OMV‐surface marker protein OmpC confirmed that isolated OMVs from both *E. coli* ^Cre^ and *E. coli* ^GFP^ show similar protein contents (Figure S2a).

Bacterial OMVs can enter host cells via several endocytosis pathways often with multiple pathways being utilized simultaneously. Previously it has been demonstrated that uptake of OMVs by epithelial cells occurs predominantly via dynamin‐dependent endocytosis (Jones et al., 2020; O'Donoghue & Krachler, 2016). To test whether we can block this typical uptake mechanism of OMVs, we microinjected purified OMVs derived from *E. coli^Cre^* into *Rosa26.tdtomato* intestinal organoids and added the dynamin GTPase inhibitor Dynasore to the culture medium (Jones et al., 2020; O'Donoghue et al., 2017). Blocking endocytosis by Dynasore significantly reduced uptake of OMVs by epithelial cells, as indicated by a significantly reduced number of organoids that displayed tdTomato‐positive cells (Figure 2d,e) providing further evidence for OMVs as relevant shuttle system for bacterial biomolecules. Moreover, we observed that the individual intensity of recombination in organoids treated with Dynasore was strongly reduced compared to the control group (Figure 2e).

### 2.3 Local induction of marker gene expression in the gut

**FIGURE 3:**
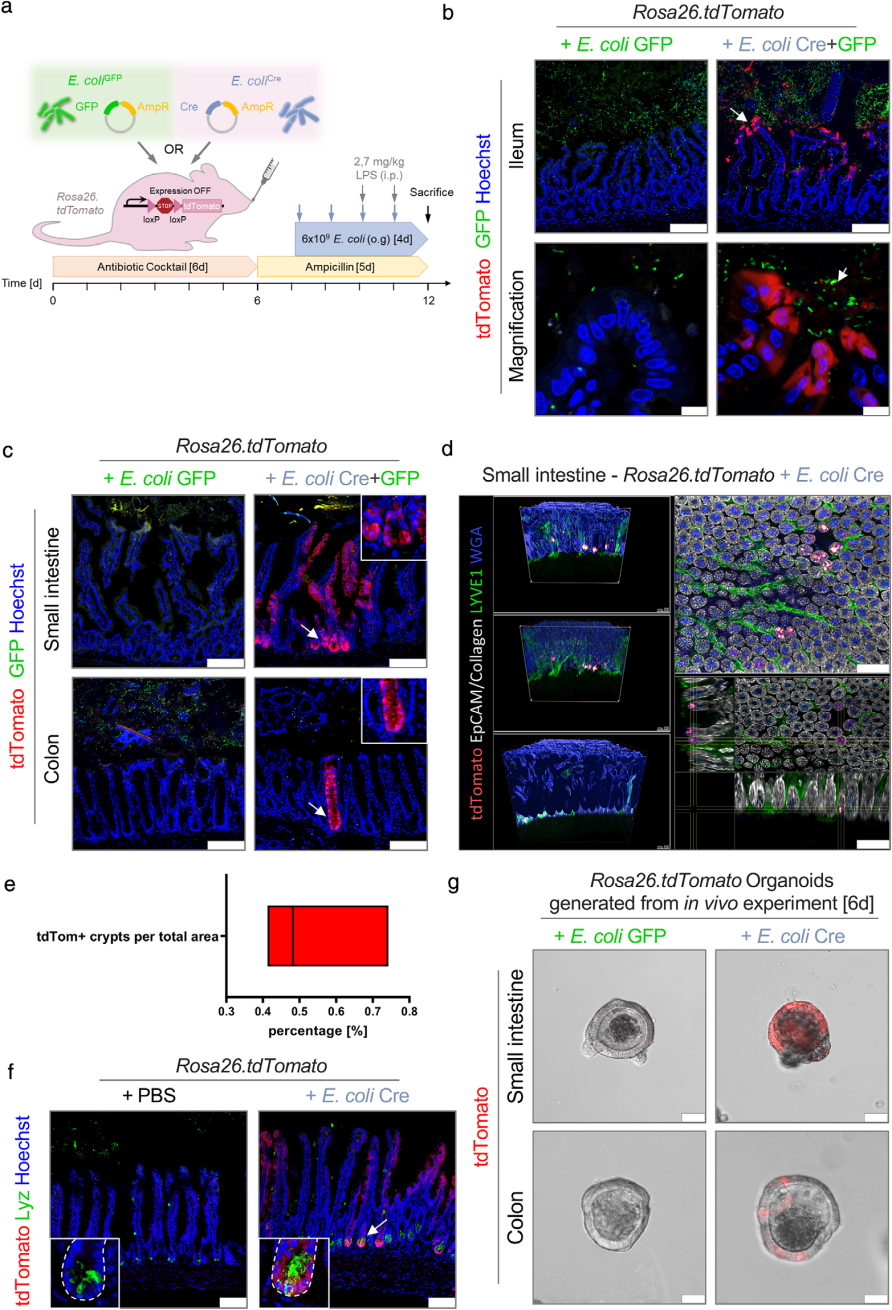
In vivo transfer of bacterial Cre to mucosal host cells. (a) Experimental set up of in vivo experiments. (b+c) Representative data derived from the intestinal tissue of *Rosa26.tdTomato* mice treated with either *E. coli* ^Cre^ plus *E. coli* ^GFP^ (*n* = 9) or with *E. coli* ^GFP^ only (*n* = 5). Experiments were performed in three independent experiments with similar results. (b) Representative confocal images of small intestinal (Ileum) cryo‐cross sections showing tdTomato‐positive epithelial cells (red) and *E. coli* ^GFP^ (green). Nuclei counterstaining with Hoechst (blue). Scale bar: 100 μm; Magnification, Scale bar: 10 μm. (c) Representative images of small intestinal and colonic cross‐sections demonstrating tdTomato‐positive cells (red) and *E. coli* ^GFP^ (green). Inset: Higher magnification of the stem cell region. Nuclei counterstaining with Hoechst (blue). Scale bar: 100 μm. (d) Volumetric reconstruction images of *Rosa26.tdTomato* intestinal mucosa treated with *E. coli* ^Cre^ showing red‐to‐white gradient with tdTomato signal in crypts (red), as well as collagen (SHG; grey), epithelial cells (EpCAM; grey), LYVE1 (green), and WGA (blue). Total analysis of 10,000 ileal crypts derived from *Rosa26.tdTomato* mice treated with *E. coli* ^Cre^ (*n* = 3). Scale bar: 100 μm. (e) Quantification of tdTomato‐positive crypts per sample area. (f) Representative immunohistochemical images of small intestinal (Ileum) cryo‐cross sections of *Rosa26.tdTomato* mice treated with *E. coli* ^Cre^ or PBS as control. Confocal pictures visualized tdTomato‐positive cells (red) and staining against Paneth cells (Lyz, Green). Nuclei counterstaining with Hoechst (blue). Scale bar: 100 μm. (g) Representative confocal images of organoids derived from the small and large intestine of *Rosa26.tdTomato* mice colonized with *E. coli* ^Cre^ (right) or as a control *E. coli* ^GFP^ (left) 6 days after isolation. Scale Bar: 50 μm. Experiments were repeated three times with similar results

To investigate the extent of microbe‐host communication via OMVs in vivo, we adapted the established model system (Ridder et al., 2014, 2015) to visualize the transfer of functional microbial biomolecules to mammalian cells within the host system (Figure 3a). To this end, *Rosa26.tdTomato* reporter mice were colonized with *E. coli* ^Cre^. Additional inoculation of the green‐fluorescent *E. coli* ^GFP^ enabled us to visually trace the location and successful colonization of the bacteria in the gut (Figure 3a,b, Figure S2b,c). Four days after bacterial inoculation, we observed Cre‐induced tdTomato signals in extended epithelial surface throughout the entire intestine of *Rosa26.tdTomato r*eporter mice harboring *E. coli* ^Cre^ but not in mice that received *E. coli* ^GFP^ only (Figure 3b and Figure S3c). The abundance of *E. coli* ^GFP^ as well as the frequency of Cre‐induced tdTomato signals in the intestinal epithelium continuously increased along the small intestine towards the terminal Ileum. Of note, in accordance with this observation, under physiological conditions there is an increasing prevalence of intestinal microbes along the small intestine with the highest density in the terminal ileum. In this region, we observed the highest incidence of tdTomato‐positive cells in areas of close proximity of *E. coli* to the intestinal epithelium (Figure 3b). Accordingly, tdTomato‐positivity was most prominent in epithelial cells at the villus tips (Figure 3b), finger‐like protrusions into the intestinal lumen and thus constantly exposed to luminal contents (Ramanan & Cadwell, 2016). Importantly, while we could observe *E. coli* ^GFP^ in close proximity to tdTomato positive cells, we could not observe GFP signals within these cells (Figure 3b). These data suggest that bacterial Cre was delivered via OMVs and not by uptake of intact bacteria. Surprisingly, using a tissue volume imaging approach, as well as confocal microscopy, we could also detect robust tdTomato signals in 0,55 % +/‐0,17 (56 out of *n* = 10,177) of analysed ileal crypts (Figure 3c–e Figure S2d; Video S1 of ileum 3D reconstruction) of *Rosa26.tdTomato* mice inoculated with *E. coli* ^Cre^, devoid of any detectable *E. coli* ^GFP^ within or in close proximity to the crypts (Figure 3b+c) and Figure S2f). Of note, this region is generally considered as a rather sterile environment (Wehkamp & Stange, 2020; Wright, 2000) due to the secretion of antimicrobial peptides including defensins and lysozyme by Paneth cells localized at the bottom of the crypts (Figure 3f) (Elphick & Mahida, 2005). Importantly, the crypt region is known to harbour in addition the intestinal stem cell reservoir and their progeny, referred to as transit‐amplifying cells (Spit et al., 2018). Interestingly, we intermittently observed tdTomato signals spanning the entire crypt‐villus axis throughout the gut, with the most abundant events detected in the small intestine (Figure 3c, Figure S2e). The fact that we observed marker gene expression in all cells of these characteristic ‘long‐lived “ribbons”’, first identified in intestinal stem cell lineage tracing experiments by Hans Clevers (Barker et al., 2007; Schuijers et al., 2014), indicated that bacteria‐derived Cre‐containing OMVs can target intestinal stem cells (Figure 3c,d and Video S1). Of note and in line with these in vivo results, we also observed an accumulation of OMVs in the stem cell areas (buds) in our organoid experiments (Figure 2c). These progenitor cells amplify and expand from the crypt, differentiate into functional intestinal epithelial cells, transit, and elongate along the crypt‐villus axis until old cells are shed at the villus tip into the lumen. After 4–5 days, the full crypt‐villus axis has been renewed and consists of recombined daughter cells (Howell & Wells, 2011; Schuijers et al., 2014; van der Flier & Clevers, 2009). To provide further evidence for the transfer of microbial functional biomolecules to intestinal progenitor cells, we generated small intestinal organoids derived from *Rosa26.tdomato* reporter mice inoculated with *E. coli* ^Cre^ (Figure S2e, according to the protocol described before in Figure 3a). In line with our in vivo results, we observed a high incidence of tdTomato‐positive organoids generated from the in vivo setting (Figure S2g). Six days after the expansion of potentially recombined intestinal stem cells in culture, including several passaging steps, we were indeed able to detect fully tdTomato‐positive intestinal organoids (Figure 3g), demonstrating a full exchange of the intestinal epithelium by recombined daughter cells (Yui et al., 2012). These data strongly support our in vivo observation that microbial OMVs can be transmitted to the sterile crypt region and thus taken up by intestinal resident stem cells, pointing towards a potential impact of transferred biomolecules on host gene regulation in target cells (Tsatsaronis et al., 2018). OMV uptake could therefore have effects that transcend multiple cellular generations.

### 2.4 Uptake of *E. coli* derived OMVs in the mucosal immune cell compartment

**FIGURE 4:**
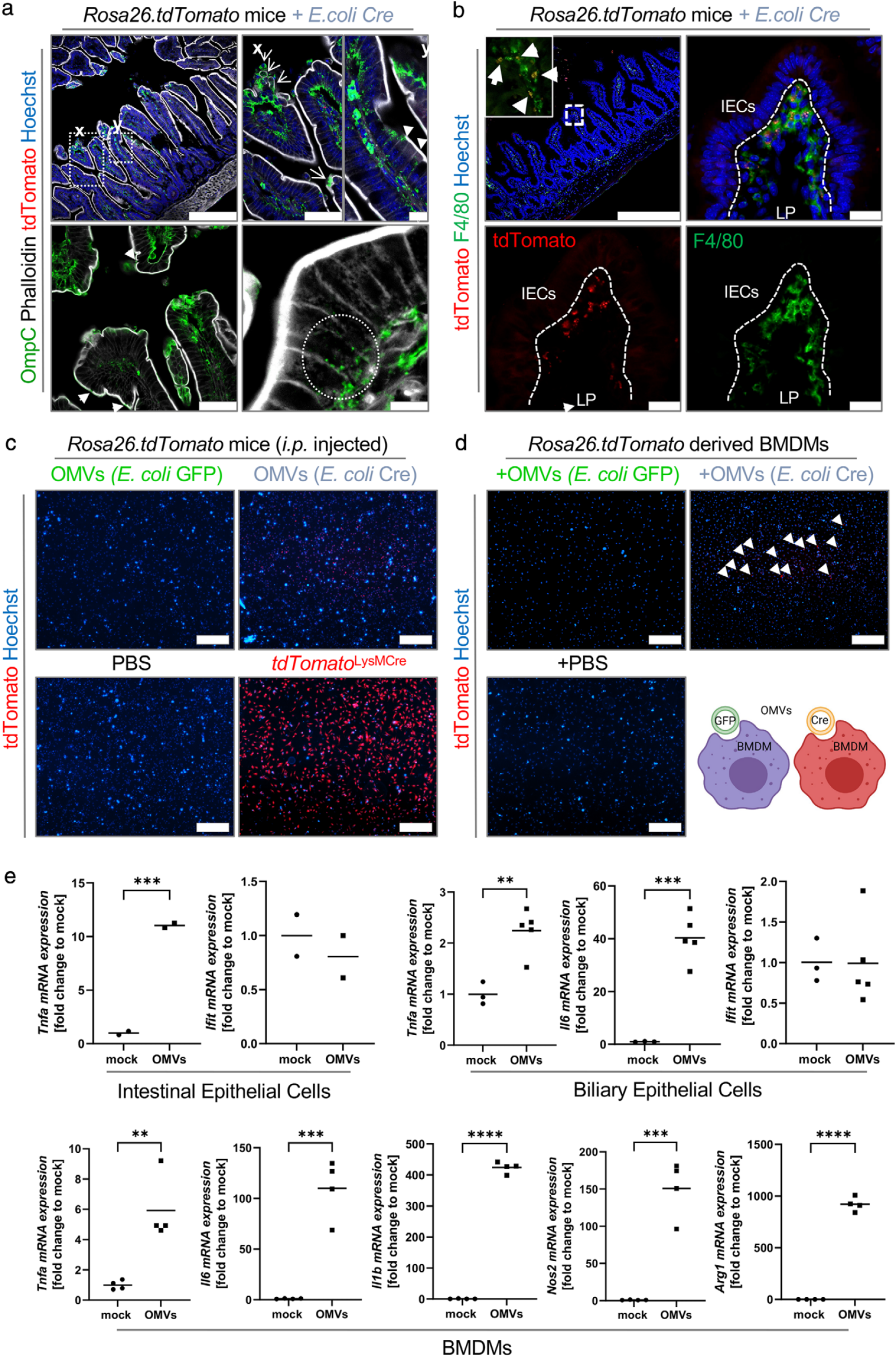
OMV uptake within the gut mucosa. (a) Representative confocal images of small intestinal (Ileum) cryo‐cross sections derived from *Rosa26.tdTomato* mice that received *E. coli* ^Cre^ and LPS stained with an antibody against OmpC to visualize OMVs and Phalloidin to stain actin filaments. Nuclei counterstaining with Hoechst (blue). Scale bar: 250 μm; Magnification, Scale bar: 75 μm and 10 μm. Arrows point towards damaged surface epithelium, arrow heads point towards OMVs within epithelial cells and at the apical surface of goblet cells. (b) Representative confocal images of small intestinal (Ileum) cryo‐cross sections derived from *Rosa26.tdTomato* mice that received *E. coli* ^Cre^ (+LPS) showing tdTomato positive macrophages. Nuclei counterstaining with Hoechst (blue). Scale bar: 250 μm; Magnification, Scale bar: 50 μm. Arrows point towards tdTomato positive macrophages. (c) Representative microscopic pictures of peritoneal macrophages isolated from *Rosa26.tdTomato* mice *i.p*. injected with 2*10^9^ particles of OMVs purified from *E. coli* ^Cre^ or *E. coli* ^GFP^ or mice treated with PBS (negative control) or isolated from *Rosa26.tdTomato*x*LysMCre (positive control)*. Nuclei counterstaining with Hoechst (blue). Scale bar: 100 μm. (d+e) Cell cultures were treated with 2*10^9^OMVs/ml. (d) Representative pictures of peritoneal macrophages isolated from *Rosa26.tdTomato* mice cultured in the presence of isolated OMVs (*E. coli* ^Cre^ or *E. coli* ^GFP^) or treated with PBS as control. Nuclei counterstaining with Hoechst (blue). Scale bar: 100 μm. (e) mRNA expression of indicated genes in intestinal epithelial cells (2D monolayer derived from murine small intestinal organoids), biliary epithelial cells (2D monolayer derived from murine liver ductal organoids) and BMDMs cultured for 8 h in the absence (mock) or presence of OMVs. Gene expression levels are shown relative to *Gapdh*. ****P < 0.0001, ***P < 0.001, **P < .01

So far, the uptake of OMVs by intestinal epithelial cells was shown indirectly by the expression of the *Rosa26.tdTomato* reporter in host cells. To directly visualize OMVs within host tissues in vivo, we stained small intestinal tissue derived from *E. coli* ^Cre^ treated *Rosa26.tdTomato* mice with an antibody specific for the bacterial surface‐protein OmpC to detect OMVs and counterstained with actin‐binding phalloidin to highlight the cellular borders of mammalian cells (Figure 4a). Intestinal tissue derived from germ‐free animals was included as a negative control to provide proof for antibody specificity (Figure S3a). OmpC signals could be detected along the whole crypt‐villus axis with a higher frequency at the surface epithelium (Figure 4a, Figure S3b,c). We could observe an accumulation of OmpC in areas of epithelial damage (Figure 4a, upper panel x) and within the apical surface of goblet cells (Figure 4a, lower panel arrows). In general, OmpC could be detected within individual epithelial cells and within cells of the underlying immune cell compartment of the gut mucosa (lamina propria) (Figure 4a, upper panel y, lower panel). The intestinal mucosa is populated by a diverse spectrum of immune cells reflecting its continuous stimulation by luminal antigens derived from various environmental factors such as the intestinal microbiota. In this context, it harbours the largest reservoir of macrophages in the body. These highly phagocytic cells are important gatekeepers to maintain intestinal homeostasis as they regulate inflammatory responses to bacteria and antigens that have crossed the intestinal epithelium. They further protect the gut mucosa against harmful pathogens and are responsible for clearing dead or senescent epithelial cells. To investigate, if OMVs are taken up by gut phagocytes, we examined the source of tdTomato positivity in the lamina propria. We could observe tdTomato‐positive macrophages with a high frequency within the upper part of the intestinal villi (Figure 4b). In line with their strategic position near the huge number of luminal bacteria, we observed a high number of tdTomato‐positive macrophages particularly directly underneath the basolateral side of the epithelial layer (Figure 4b, high magnification), the area where we also observed a high density of OMVs (Figure 4a, upper panel x, lower panel circled area). Again, we could not detect GFP positive signals within or in close proximity to these tdTomato positive cells, indicating the absence of bacteria within this area. These data indicate that OMVs can cross the intestinal epithelial barrier and are taken up by immune cells locally in the gut.

In the next step, we were interested whether OMVs also target macrophages that reside outside the gut wall. To test if OMVs can transfer functional Cre to macrophages outside the gut, we adapted our experimental setting and injected (*i.p*.) purified OMVs isolated from *E. coli^Cre^* or *E. coli^GFP^* (as a control) in *Rosa26.tdtomato* mice. Similarly, as observed within the gut mucosa, we could detect a high number of tdTomato‐positive peritoneal macrophages, immune cells that reside in the peritoneal cavity, a fluid‐filled space located between the mucosal wall and the organs found in the abdomen (Figure 4c). To provide further support of uptake of OMVs by phagocytes, we cultured primary bone marrow derived macrophages (BMDM) derived from *Rosa26.tdtomato* mice in the presence of purified OMVs derived from *E. coli^Cre^* or *E. coli^GFP^*. Again, we observed tdTomato positive cells in cultures that contained Cre vesicles only (Figure 4d). These data add compelling evidence to previous in vitro and in vivo findings with labelled OMVs, suggesting that OMVs can not only cross the mucus layer to reach the intestinal epithelium, but even surpass the epithelial border, delivering bacterial antigens to the underlying immune system, which might trigger intestinal inflammation (Hickey et al., 2015; Schwechheimer & Kuehn, 2015). To investigate the immune modulatory properties of the OMVs on the host, we exposed intestinal organoids (as first line of defence), BMDMs (second line of defence) and liver ductal organoids (as tissue resident liver cells) to OMVs. We could observe a pronounced inflammatory response including the expression of cytokines such as *Tnfa* among all analysed host cells (Figure 4e, Figure S3d). In summary, these data further highlight the dynamics of host‐microbe interaction via OMVs in the gut.

### 2.5 Transfer of bioactive molecules by OMVs to multiple organs

**FIGURE 5:**
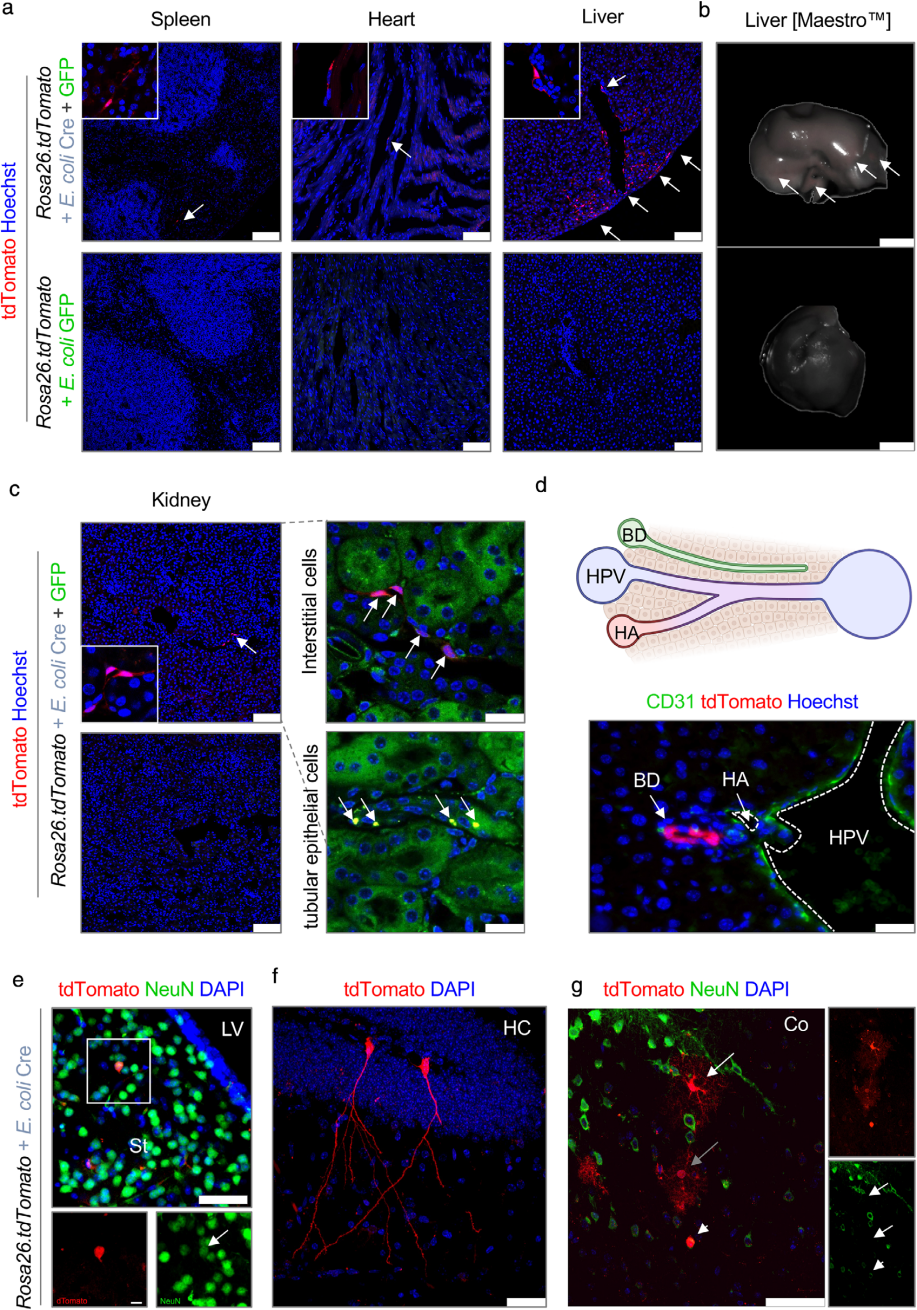
Bacterial OMVs transfer functional Cre mRNA to multiple organs. (a–g) Representative data derived from different organ tissue of *Rosa26.tdTomato* mice treated with either *E. coli* ^Cre^ plus *E. coli* ^GFP^ (*n* = 9) or with *E. coli* ^GFP^ only (*n* = 5). Experiments were repeated 3 times with similar results. (a) Representative confocal images of different organ cross sections of spleen, heart and liver showing tdTomato‐positive cells (red). Nuclei counterstaining with Hoechst (blue). Scale bar: 100 μm. (b) Representative Maestro *ex vivo* images of the liver of *Rosa26.tdTomato* mice treated with *E. coli* ^Cre^ (plus *E. coli* ^GFP^) or *E. coli* ^GFP^ as control analyzed via multi‐spectral separation. Scale bar: 50 mm. (c) Representative confocal images of kidney cross‐sections showing tdTomato‐positive cells (red). Tubular epithelium shown in green. Nuclei counterstaining with Hoechst (blue). Scale bar: 100 μm. (d) Representative pictures of tdTomato positive biliary epithelial cells in liver cross sections. Scale bar: 50 μM. Arrows point towards tdTomato‐positive cells. HPV: hepatic portal vein; HA: hepatic artery; BD: bile duct. Scale bar: 25 μm. (e‐g) Representative confocal images of immunohistochemical staining of brain cross‐section showing tdTomato‐positive cells (red) and staining against NeuN (e,g) (Green, Neuron). Nuclei counterstaining with DAPI (blue). Scale bar: 50 μm (Upper image), 10 μm (Lower image). LV: lateral ventricles; St: striatum; HC: hippocampus; Co: cortex

So far, we showed OMV‐mediated transfer of functional biomolecules along the gastrointestinal tract into intestinal epithelial cells, including epithelial progenitor cells, as well as cells of the mucosal immune cell compartment. However, gut microbes and gut‐derived microbial products have not only been associated with an influence on the function of the gastrointestinal tract but also on organs without direct contact to the intestinal microbiota, for example, via translocation of microbial products into the peripheral circulation (Brenchley & Douek, 2012). Accordingly, previous studies have detected high levels of exogenous bacteria‐derived biomolecules such as microbial RNA, bacterial toxins or peptidoglycan in human biofluids (Godoy et al., 2018; Veziroglu & Mias, 2020). To test whether OMVs can cross the intestinal barrier and enable protected distribution of microbial‐derived bioactive molecules towards peripheral organ systems, we further expanded our analysis to peripheral organs (Figure 5). In line with the strong impact of intestinal microbes on physiology of various organs, we observed tdTomato‐positive cells in counterstained cryo‐cross‐sections of all analysed organs including the spleen, heart, liver, kidney, and brain (Figure 5a‐e). We even observed a slight elevation in tdTomato signal strength in the liver of *E. coli* ^Cre^ treated *Rosa26.tdTomato* mice via whole‐organ analysis using the Maestro imaging system (Figure 5b, right). Of note, we could not observe tdTomato‐positive cells in the lung, which might be due to too faint signals. Interestingly, in the kidney we could observe tdTomato‐positive cells within the tubular renal interstitium (which harbours immune cells of the kidney) and tubular system (tubular epithelial cells) which originate in the kidney (Figure 5c). In the liver, we observed that uptake of OMVs was most prominent within the portal triad, which includes hepatic artery, hepatic portal vein as well as small bile ductules (Total number: 6,4 (+/‐2,96) tdTomato positive cells per liver lobe cross section) (Figure 5d, Figure S4a). Within this area, we could detect uptake of OMVs by endothelial cells, biliary epithelial cells as well as hepatocytes. The fact that tdTomato positive cells were most present in this area, further support the notion that OMVs are delivered via the bloodstream, since the hepatic portal vein is a blood vessel that carries blood from the gastrointestinal tract, gallbladder, pancreas and spleen to the liver. Importantly, in none of the analysed organs, we could observe GFP signals, indicating that intestinal microbes were not able to cross the intestinal barrier. In the brain, we detected tdTomato^+^ NeuN^+^ double‐positive neurons in mice receiving *E. coli* ^Cre^ (Total number: 14.2 tdTomato positive neurons per hemisphere ± 10.2 SD; *n* = 6) but none in mice that received *E. coli* ^GFP^ only (*n* = 4) (Figure 5d). Neurons that received functional Cre via bacterial OMVs were predominantly located in the striatum (Figure 5e) and to a lesser extent in the cortex and hippocampus (Figure 5f). Of note, we could also detect a small number of NeuN‐negative glial cells (Figure 5g). To exclude the possibility of non‐labelled bacteria crossing the epithelial barrier to reach distant organs, which in turn might trigger tdTomato expression locally, we performed Fluorescence in situ hybridization (FISH) with a universal bacterial oligonucleotide probe EUB‐338. While we could observe FISH‐positive bacteria signal in the small and large intestine of *Rosa26.tdtomato* mice inoculated with *E. coli^Cre^*, we could not observe FISH‐positive signals in the spleen, liver, brain, heart, or kidney despite the observed tdTomato‐positive signals (Figure S4b). These data strongly implicate that OMVs can shuttle bacterial derived bioactive molecules from the intestine across various barriers, including the intestinal barrier as well as the blood‐brain barrier, to peripheral organs. The fact that bacterial OMVs can overcome the blood‐brain‐barrier is in line with our previous observations of EVs generated by blood cells and released into the peripheral circulation that enter the brain (Kur et al., 2020; Ridder et al., 2014).

In summary, by taking advantage of a newly established in vivo model, we were able to demonstrate—for the first time—inter‐kingdom transfer of functional bioactive molecules between gram‐negative bacteria and individual mammalian host cells including stem cells. Our findings suggest that transfer of microbial bioactive factors hold the potential to induce not only short‐term effects within intestinal epithelial cells, but also inheritable changes by targeting intestinal stem cells. Together, our findings substantially extend our understanding on the scope of microbiota‐host‐interactions.

## 3 DISCUSSION

Shedding of extracellular vesicles (EVs) represents a universal mechanism for inter‐ and intra‐kingdom communication among all domains of life: eukaryotes, bacteria and archaea (Kalluri & LeBleu, 2020). While, initially the secretion of EVs was described as a mechanism of selected elimination of proteins, lipids or RNAs from cells or bacteria, nowadays they are also considered as a novel mode of inter‐ and intra‐kingdom communication. The tremendous progress in our understanding of the functional significance of the microbiome for human health and disease thus has triggered enormous interest in the biology of microbial‐derived EVs.

However, insights into the relevance of host‐microbe interaction and the impact of microbes on the pathophysiology of the host, has so far been largely correlative. Indeed, support of this process derived primarily from experiments based on the comparison of mice with altered, reduced or completely depleted microorganisms. Going one step further, recent studies have started to characterize the extend of inter‐kingdom communication via EVs released by bacteria (Jones et al., 2020). In these studies, OMVs were labeled in vitro, and their distribution was analyzed after their injection in vivo. While these important studies provided some support for a role of OMVs in host‐microbe communication, they nevertheless have significant limitations. Labelling of OMVs does not allow to discriminate functional uptake from other processes, in particular degradation, which is probably the most common fate after uptake. Moreover, OMVs used in these studies were often labelled *ex vivo* (Choi et al., 2015), possibly introducing bias based on the purification techniques, generating unspecific fluorescent particles, and altering their biological properties. Furthermore, in many of these studies, OMVs were introduced into mice by injection either in the peritoneum (Jang et al., 2015) or directly into the affected organ (Han et al., 2019), which neither reflects their physiological distribution nor their quantity over time.

In sharp contrast to previous studies that investigated this question, the results of our study demonstrate for the first time in a proof‐of‐concept study the trafficking, and biodistribution of bioactive molecules by OMVs to individual eukaryotic cells in vivo. By using the Cre‐LoxP system, we were able to demonstrate in vivo functional transfer of OMV cargo to their target cells. Thus, our study identifies OMVs as far‐reaching horizontal shuttle systems that serve as a communication tool to maintain and modulate microbe‐host interactions.

Pinpointing individual cellular targets of bacterial OMVs will allow an unprecedented level of mechanistic understanding of how the microbiome might influence cellular function. In line with our results, previous studies showed that humans typically carry high levels of extracellular RNA (exRNA) including mRNAs, microRNAs and other small RNAs that are contained in EVs in their biofluids. Interestingly some of these fluids, such as saliva, have high levels of exogenous sequences that can be traced to bacteria (Yeri et al., 2017), which is not surprising given the fact that there are estimated to be up to 10 times more microbes than host cells in the human body. In addition, OMVs have been identified in a variety of infected host tissues (Craven et al., 1980; Stephens et al., 1982; Vidakovics et al., 2010). Moreover, increasing evidence supports the notion that pathogens use OMVs to deliver virulence factors into host cells at local and distal sites, which might be associated with altered immune responses (Ellis and Kuehn, 2010). In line with this assumption, we show that bacterial‐host communication via OMVs is not limited to the transfer of bacterial information to the intestinal epithelium, but rather extends to a widespread distribution to multiple organs and cell types. The fact that bacterial OMVs can deliver and spread bioactive molecules protected from enzymatic degradation over a long distance throughout the body, far away from their parental bacteria in the gut and even across the blood‐brain‐barrier to neurons, provides a substantial advantage in contrast to other major bacterial secretion systems. It should be kept in mind that the pattern of cells active in taking up OMVs that we observe histologically very likely does not solely reflect biodistribution of the OMVs, but rather is a combination of the physiological state of such cells and the bacterial species of origin for the OMVs as we could show for the transfer of EVs from blood to brain (Kur et al., 2020). In this respect, it is interesting to speculate how the extent and distribution of OMV uptake would be when using more physiological bacterial species, perhaps in combination with specific models of injury or disease.

Work reported by other groups has demonstrated effects that OMVs can exert on host physiology. In this context, a study of the Stappenbeck lab could demonstrate that orally gavaged OMVs derived from *Bacteroides thetaiotaomicron* (*B. thetaiotaomicron*) can efficiently cross the intestinal barrier and access the host´s immune system, in particular macrophages in a sulfatase‐dependent process (Hickey et al., 2015). They further reported that this was sufficient to cause inflammation in the gut. In line with that study, we also observed robust OMV uptake by mucosal macrophages both in vivo and in vitro and OMVs induced a marked proinflammatory response in these phagocytic cells. OMVs contain multiple pathogen‐associated molecular patterns (PAMPs) and can thus easily be detected and internalized by phagocytes, which likely contribute to account for the high abundance of OMV uptake detected for macrophages. In sharp contrast, OMVs derived from *Bacteroides fragilis* have been described as regulatory modulators of immune responses that can prevent intestinal inflammation (Chu et al., 2016). In this study, the authors could demonstrate that OMV uptake by dendritic cells induces regulatory T cells. This was further supported by another study demonstrating the impact of *B. thetaiotaomicron* derived OMVs on T cell function (Wegorzewska et al., 2019). While both studies were focused on the effect of OMVs on the mucosal immune system, another study demonstrated that intraperitoneal administration of *E. coli* derived OMVs was followed by an infiltration of neutrophils into the lung (Lee et al., 2018). In vitro studies by this group further demonstrated that exposure of endothelial cells to OMVs was associated with a release of IL‐8/CXCL1 which might recruit neutrophils to the lung. Interestingly, in line with this observation, our study firmly demonstrated uptake of OMVs by endothelial cells (in the liver) supporting this model. These previous studies demonstrated that microbiota‐derived OMVs can both promote or prevent inflammation in the gut and even within distant organs. Our data suggest that microbe‐host communication is highly abundant in mucosal tissues of the gut and that inflammatory signals are important initiators in fostering translocation of OMVs to distant organs via the blood stream. Consistent with our findings, results of several prior studies detected bacterial RNA in human blood samples by 16S rRNA sequencing that are likely derived from OMVs (Castillo et al., 2019; Nikkari et al., 2001; Païssé et al., 2016; Turnbaugh et al., 2007).

In summary, the emerging field of EVs, including a variety of microbiota‐derived EVs such as membrane vesicles, outer membrane vesicles and apoptotic bodies, has gained increasing interest with respect to its pharmacological potential. Accordingly, EVs take centre stage in the pipeline as novel smart drug carriers for targeted drug delivery, biomarkers in diagnostic and early cancer detection as well as a promising tool for vaccine development. Therefore, our study provides fundamental new information about the cellular targets of OMVs throughout the host system, which is of particular interest to better estimate the biological range of such novel therapies.

## 4 MATERIAL AND METHODS

### 4.1 Animals and housing

C57BL/6J wild‐type mice (Stock No: 000664) and B6.Cg*‐Gt(ROSA)26Sor^tm9(CAG‐tdTomato)Hze^*/J (*Rosa26*.*tdTomato*) reporter mice (Stock No.: 007905)^27^ were obtained from the Jackson Laboratory. *Rosa26*.*tdTomato* mice carry a *loxP*‐flanked STOP cassette preventing transcription of a CAG promoter‐driven fluorescent protein variant (*tdTomato*)–all inserted into the *Gt(ROSA)26Sor* locus. Following Cre‐mediated recombination cells of these reporter mice express robust tdTomato fluorescence. Mice were housed and bred in specific pathogen‐free conditions in animal Research Facilities located at the San Francisco Veterans Affairs Medical Centre, and at the Kussmaul‐Science Campus of the University clinic Erlangen. Mice were routinely screened for pathogens according to FELASA guidelines. Animal experiments were approved by the Institutional Animal Care and Use Committee at the VA Medical Centre in San Francisco, and the Regierung von Unterfranken.

### 4.2 Animal protocols

All animal experiments were performed under sterile conditions including treatment and housing. Mice were housed in individually ventilated sterilized cages using the IsoCage P‐Bioexclusion System with HEPA Filter only receiving autoclaved mouse feed and water.

For a reduction of the endogenous microbiota *Rosa26*.*tdTomato* mice were pretreated with an antibiotic cocktail (Ampicillin 1 mg/ml, Vancomycin 0,5 mg/ml, Metronidazole 1 mg/ml; Neomycin 1 mg/ml plus Splenda 20 mg/ml) via drinking water for 6 days (Day 1–6). On day 7 antibiotic treatment was switched to selection antibiotic treatment with Ampicillin (1 g/L) via drinking water to generate a selective ecological niche for *E. coli* ^Cre^ and *E. coli* ^GFP^ to prevail against the endogenous microbiota. On day 8 to 11, mice were daily inoculated via oral gavage with either Ampicillin‐resistant *E. coli* ^Cre^ [3 × 10^9^ *E. coli* ^Cre^ (plus 3 × 10^9^ *E. coli* ^GFP^ intial dose, day 8)] or *E. coli* ^GFP^ [6 × 10^9^ *E. coli* ^GFP^] as control. As quality check, bacterial abundance (CFU/g stool) was routinely verified from faecal samples on day 0, 6 and 12 on LB‐Agar plates plus/minus Ampicillin (100 mg/L). In order to enhance gut barrier permeability facilitating bacterial OMV transmigration, mice were injected twice intraperitoneally with 2,5 mg LPS per kg body weight (Day 10 and 11) as previously described by (Günther et al., 2015). On day 12 mice were sacrificed and organs were harvested or processed for further analysis.

### 4.3 Bacterial strains and growth conditions

*E. coli* ^Cre^ ‐ *Escherichia coli* DH5α with Cre vector construct containing Cre recombinase plus Ampicillin resistance gene (Addgene; plasmid # 62730) (D'Astolfo et al., 2015), kindly provided by Niels Geijsen.

*E. coli* ^GFP^ ‐ *Escherichia coli* GFP (ATCC 25922GFP) with GFP vector construct pUCP18MCSgfpmut3 containing fluorescent GFP plus Ampicillin resistance gene (Barbier & Mittar, 2014; Choi & Schweizer, 2006).

### 4.4 Bacteria culture

*E. coli* ^Cre^ and *E. coli* ^GFP^ were cultured in autoclaved LB‐medium (25 g/L (Luria/Miller); Roth) plus selection antibiotic Ampicillin (100 mg/L). For solid media, 15 g/L Bacto Agar‐Agar (Roth) was added. Incubation of liquid cultures of *E. coli* was carried out at 37°C under shaking in baffled flasks (150 rpm) with Isopropyl β‐ d‐1‐thiogalactopyranoside (IPTG). Overnight cultures were used to inoculate fresh media to an OD_600_ of ∼ 0.1, bacteria were incubated at 37°C until the exponential growth phase was reached (OD_600_ ∼ 1.0). Bacteria cultures were harvested by centrifugation (10 min, 4°C, 4000×g) and were further processed as described above or below.

### 4.5 Outer membrane vesicle purification

Supernatant from *E. coli* culture (800 ml) was 0.22‐ μm filtered after centrifugation to remove larger particles/ bacteria and mixed with polyethylene glycol (PEG) 8000 (Rotipuran, 0263.1, Roth) solution 1:5 for precipitating vesicles during an overnight incubation at 4˚C. Samples were centrifuged at 1500×g for 30 min at 4˚C and pellets were resuspended in 10 mM HEPES buffer for an ultracentrifugation step (Sorvall WX Ultra Series 80; Thermo Fisher Scientific) at 100,000×g for 90 min. The resulting pellet was resuspended in 10 mM HEPES and stored at 4°C.

OMVs concentrated by PEG precipitation were also further purified by OptiPrep iodixanol density gradient ultracentrifugation as previously reported (Klimentová & Stulík, 2015). To this end, OMV PEG pellets were first resuspended in 1 ml of PBS and mixed with 2 ml of 60% iodixanol (Sigma). The mixture was placed under a 9 ml OptiPrep density gradient (5%, 10%, 20% w/v iodixanol) and spun at 100,000 ×g for 18 h at 4°C (SW 40 Ti rotor) (Duong et al., 2019). Afterwards, 12 1 ml fractions were collected starting from the top of the tube. Fractions 7–9 of the gradient were mixed and dialyzed in PBS with the Slide‐A‐Lyzer Dialysis Device (Thermo Fisher Scientific) with a 10,000 MW cut off, 15 ml capacity. The OMVs were initially suspended in 3 ml and after dialysis the volume increased to 6 ml. Protein concentrations were at 0.613 mg/ml for Cre‐OMVs (total amount 3.68 mg) and 0.608 mg/ml for GFP‐OMVs (total amount 3.65 mg). Subsequently, OMVs were further diluted to a volume of 40 ml in PBS and pelleted by ultracentrifugation with 100,000xg for 18 h at 4°C (SW 28 Ti rotor). The OMV pellets were then resuspended in 1 ml of PBS and subjected to size and concentration measurement by NanoSight LM14 (Malvern Instruments, Westborough, MA) performed with a 488‐nm detection wavelength. The analysis settings were optimized and kept identical for each sample, with a detection threshold set at three, three videos of 1 min each were analysed to give the mean, mode, median, and estimated concentration for each particle size. Samples were diluted in 1:100 PBS and measured in triplicates. Data were analysed with the NTA 3.3 software. Particle numbers were 9,58E+10/ml for the OMV‐Cre pooled fractions F7‐F9 (26E+6 particles per μg protein) and 1,17E+11/ml for the equivalent OMV‐GFP stock (32E+6 particles per μg protein). All the OMV samples were used fresh (less than a month after isolation) and stored at 4°C.

### 4.6 Electron microscopy

Vesicle preparations were fixed and stained as described (Bouchareychas et al., 2020) with minor alterations. The uranyl‐oxalate (step 6) was substituted with 2% of uranyl‐acetate, and the ratio of methylcellulose to uranyl‐acetate was changed to 1% each (step 7). Grids were analysed using Tecnai Spirit BioTWIN electron microscope (FEI, Netherlands) at 120 kV. Pictures were taken with an eagle bottom‐mount CCD camera and processed with ImageJ (NIH).

### 4.7 Intestinal organoid culture

Small intestinal and colon organoids were isolated from the mouse intestine and cultured for a minimum of 6 days as described earlier (Günther et al., 2011). In brief, crypts were isolated by incubating pieces of the small intestine or colon of 8‐ to 12‐week‐old C57BL/6J mice rolling in isolation buffer (Phosphate buffered saline (PBS), 2 mM EDTA; 4°C) and consequent repetitive steps of vortexing, washing (1xPBS) and centrifugation (5 min, 200×g). Finally, crypts were transferred in 25 μl Corning Matrigel into 48 well plates, solidified (10 min, 37°C) and then covered with 300 μl intestinal culture medium. Medium for small intestinal organoids contained [Advanced DMEM/F12 (Invitrogen), containing HEPES (10 mM, PAA), GlutaMax (2 mM, Invitrogen), Penicillin (100 U/ml, Gibco), Streptomycin (100 μg/ml, Gibco), B27 Supplement 1x (Invitrogen), 1 mM N‐acetylcysteine (Sigma‐Aldrich), murine EGF (50 ng/ml, Immunotools); R‐spondin and recombinant murine Noggin was added equivalent to recombinant human R‐spondin (1 μg/ml, R&D Systems), and recombinant murine Noggin (100 ng/ml, Peprotech). For colon organoids IntestiCult Organoid Growth Medium (Mouse) (STEMCELL Technologies) was used. To both intestinal organoid culture media extra WNT3a (0.25 nM, U‐Protein Express BV) was added to promote additional stem cell expansion.

### 4.8 Liver ductal organoid culture (biliary organoids)

Biliary organoids were isolated from the mouse liver as previously described with slight modifications (Broutier et al., 2016 **;** Chusilp et al., 2020). In brief, liver tissue was harvested from healthy of 8‐ to 12‐week‐old C57BL/6J mice. Intrahepatic bile duct fragments were isolated from the liver by digestion using a tissue dissociation cocktail which includes Collagenase IV (0,125 mg/ml, Worthington) and Dispase II (0,125 mg/ml, Invitrogen) in Dulbecco's Modified Eagle's medium/nutrient mixture F‐12 (DMEM/F‐12) containing 1% BSA and 1% Penicillin (100 U/ml, Gibco), Streptomycin (100 μg/ml, Gibco). Intrahepatic bile duct fragments were then embedded in Corning Matrigel in a 48‐well plate. Medium for ductal organoids (ductal expansion medium) contained [Advanced DMEM/F12 (Invitrogen), containing HEPES (10 mM, PAA), GlutaMax (2 mM, Invitrogen), Penicillin (100 U/ml, Gibco), Streptomycin (100 μg/ml, Gibco), B27 Supplement 1x (Invitrogen), 1 mM N‐acetylcysteine (Sigma‐Aldrich), Gastrin (10 nM, Sigma‐Aldrich), recombinant murine EGF (50 ng/ml, Immunotools); recombinant human FGF10 (100 ng/ml, Peprotech), recombinant human HGF (HEK293 derived) (50 ng/ml, Peprotech), R‐spondin and recombinant murine Noggin was added as tested conditioned medium equivalent to recombinant human R‐spondin (1 μg/ml, R&D Systems). For the first 4 days after isolation, ductal organoids were maintained in liver isolation medium, which is the ductal expansion medium supplemented with 25 ng/ml recombinant human Noggin or 5% (vol/vol) Noggin‐conditioned medium, 30% (vol/vol) Wnt3a‐conditioned medium and 10 μM Rho kinase (ROCK) inhibitor (Y‐27632).

In some experiments, organoids were microinjected with either live bacteria or purified OMVs from *E. coli* ^Cre^ or *E. coli* ^GFP^. Z‐stack images were obtained using the confocal fluorescence microscope (LEICA TCS SP5 II) together with the LEICA DFC360 FX camera and the imaging software ‘LAS X’ (Leica).

### 4.9 3D organoid derived monolayer

Adult mouse small intestinal organoids or biliary organoids were grown for a minimum of 12 days as 3D organoids under culture conditions listed above. Intestinal organoids were cultured for 4 days on 50% L‐WRN conditioned medium to increase the number of stem cells. Biliary organoids were then dissociated into single cells by incubating in a digestion solution (TrypLE Select (Gibco), 0.5 M EDTA (Sigma‐Aldrich), 10 μM Rock Inhibitor Y‐27632 (R&D Systems) solved in PBS) and seeded onto 48 Well plates (Corning) pre‐coated by 10% Matrigel (Corning) for 20 min. Intestinal organoids were dissociated into single cells by digesting in 10% TrypLE Select (Gibco) and seeded onto 48 Well plates (Corning) pre‐coated by 3,3% Matrigel (Corning) for 2 h (∼20,000 cells were seeded per well). Intestinal 2D monolayer were cultured in IntestiCult Organoid Growth Medium (Human) (STEMCELL Technologies) with added 10 μM Rock Inhibitor Y‐27632 (R&D Systems). Ductal monolayer were cultured in ductal expansion medium. Initially, intestinal organoids were submerged in 150 μL 50% L‐WRN media with 10 μM Rock inhibitor Y‐27632 (R&D Systems).

### 4.10 BMDM isolation and stimulation

Murine BMDM were obtained as described previously (Bouchareychas et al., 2017). Briefly, bone marrow cells were flushed from the tibia and femurs of 8‐ to 12‐week‐old C57BL/6J mice. Cells were cultured in Dulbecco's Modified Eagle's Medium (Corning) supplemented with 10% fetal bovine serum (GIBCO), 1% GlutaMax (GIBCO), and 1% penicillin‐streptomycin (GIBCO) and differentiated with 25 ng/ml mouse M‐CSF (Peprotech) for 6 days. For OMV exposure experiments, BMDM were dispensed into 12‐well culture plates (Corning) at a concentration of 3 × 10^5^ cells/well and stimulated with OMVs for 24h at a concentration of 2 × 10^9^ particles per ml.

### 4.11 Blocking experiments

Small intestinal organoids derived from *Rosa26.tdTomato* mice were grown for 7 days and then microinjected (*n* = 200/well) with purified OMVs derived from *E. coli^Cre^* using a Pneumatic Pico Pump PV820 and a World Precision Instrument. Dynasore was added into the organoid culture medium 60 min prior to OMV microinjection in an 80 μM concentration (Dynamin Inhibitor I, Dynasore Sigma‐Aldrich).

### 4.12 Histology and immunohistochemistry

To fixate tdTomato in the tissue, harvested organs were incubated in ROTIHistofix 4% for 24h at room temperature (RT), then transferred to 30% sucrose solution and incubated 24h at 4°C. Tissue was embedded and sectioned in cryo‐mounting medium (Sakura OCT) at Leica CM3050S Kryostat. Immunofluorescence staining was performed using rabbit anti‐Lysozyme (Dako, Catalogue A0099, 1:2000), rabbit anti‐OmpC (biorbyt, orb6940, 1:1000) with goat anti‐rabbit‐biotinylated (Dianova, Catalogue 111‐065‐144) with Streptavidin Protein‐DyLight 550 system and mouse anti‐NeuN (Millipore, Catalogue MAB377, 1:300) with Alexa Fluor 647 goat anti‐mouse (Invitrogen, Catalogue A21235). In some experiments Paneth and goblet cells were stained with Ulex europaeus agglutinin I (UEA‐1) (Vector laboratories, Cat. No.: FL‐1061). Nuclei counterstaining on tissue‐sections was performed using Hoechst 33342 (Invitrogen) or DAPI (Sigma‐Aldrich). Images were obtained using a confocal fluorescence microscope (LEICA TCS SP5 II) together with the LEICA DFC360 FX camera and the imaging software ‘LAS X’ (Leica) or a confocal inverted microscope (Nikon Eclipse TE2000‐E) and NIS‐elements imaging software (version 4.13.05) and ImageJ (https://imagej.nih.gov/ij/).

### 4.13 *Ex vivo* organ imaging via Maestro EX imaging system

Following Fixation in ROTIHistofix 4% for 24h (RT) and incubation in 30% sucrose solution for 24h at 4°C harvested organs were fully imaged using the Maestro EX in vivo imaging system (Ecelitas Technologies, Fremont, CA, USA) including CRI MO80285‐PFC (Cambridge Res.&Instr., Woburn, MA, USA). For imaging of tdTomato fluorescence (excitation/ emission maxima at 554 nm/ 581 nm) the Green Maestro Filter Set (M‐MSI‐FLTR‐GREEN ‐ Excitation 503/555 nm and Emission filter for 580 nm longpass; Acquisition Setting: 550–800 nm in 10 nm steps) was used. For GFP imaging (excitation/ emission maxima at 484 nm/ 510 nm) the Blue Maestro Filter Set (M‐MSI‐FLTR‐BLUE ‐ Excitation 445/490 nm and Emission filter for 515 nm longpass; Acquisition Setting: 500–720 nm in 10 nm steps) was used. Overlay images were generated with the enclosed Maestro 2.6.0 software.

### 4.14 Tissue volume imaging

Intestinal samples were harvested, flushed with PBS, cut open along the mesenteric base and fixated overnight in 1% PFA/PBS (FisherScientific) at 4°C. Samples were blocked and permeabilized overnight at RT (BD Cytoperm; BD Bioscience), 10% rat serum (Sigma), 100 mM glycine (Sigma) and stained with following antibodies/reagents: LYVE1 eFluor 570 (ThermoFisher, Catalogue 41‐0443‐82), wheat germ agglutinin Alexa Fluor 647 (ThermoFisher, Catalogue W32466), EpCAM Alexa Fluor 594 (BioLegend, Catalogue 118222). Optical clearing was achieved using the Ce3D protocol (Li et al., 2017). Volume imaging was performed with sequential 1‐photon/confocal and 2‐photon excitation, using a Zeiss LSM 780 microscope with internal and external non‐descanned GaAsP detectors and ZEN black software (Zeiss). Images were taken with a 20x water immersion objective (NA 1.0, Zeiss) as tiles at a resolution of 1024 × 1024 (16bit) in stacks with a slice distance of 2 μm over a z‐range of ∼ 500 μm tissue volume in total. 1P‐ and 2P‐stacks were computationally aligned using ZEN blue 3.2 (Zeiss), and multiplex volumetric reconstruction and visualization were performed in IMARIS 9.3 (Bitplane). Quantifications included the following parameter: sample dimension, sample area, total dimension, total area, number of crypts detected within the sample area, number of crypts per total area, number of tdTomato positive crypts per total area, % of tdTomato positive crypts over the total area. An average total area of 8276908 μm^2^ (standard deviation 200462) was recorded in three dimensions per whole‐mount sample. Differentiation between tdTomato‐positive and ‐negative crypts and their quantification was performed using the IMARIS algorithm to generate surface objects with apical crypt nuclei as seed points for automatic volume segmentation of the surrounding crypt epithelium, resulting in an average of 3325 crypts per sample (standard deviation 171). Each crypt was converted into a single isosurface object and the mean intensity of the tdTomato fluorescence channel was used to distinguish positive from negative crypts for subsequent automated quantification.

### 4.15 Gene expression

RNA was purified from *E. coli* ^Cre^ and *E. coli* ^GFP^ derived OMVs and whole bacteria lysates using Qiagen RNeasy Micro Kit, according to the manufacturer's instructions. Bacteria were lysed in lysozyme‐TE buffer (15 mg/ml lysozyme) and proteinase K (1 mg/ml), OMVs were lysed directly in RLT buffer. For RNA protection assay, OMVs were mixed with 0.075% Triton X‐100 and/or 0.4 mg/ml RNase AT/1 Mix (Thermo Fisher Scientific) and incubated at 37°C for 20 min before RNA extraction. cDNA synthesis was performed using SuperScript III Reverse Transcriptase (SIII‐RT) (Invitrogen) and random Primers (Fermentas). Amplification was performed on a MyiQ Real‐Time PCR Detection System (BioRad, Germany) using an ABsolute qPCR‐mix and SYBR Green (ThermoFisher Scientific, Germany). PCR products were visualized on a 3% agarose gel.

Cre‐primer sequences: fwd: 5′‐GCCTGCATTACCGGTCGATGCAACGA‐3′; rev: 5′‐GTGGCAGATGGCGCGGCAACACCATT‐3′ (product length 726 bp). Full length: fwd: 5′‐ATTCATCGGGTCTGGTTCCG‐3′; rev 5′‐ATCGCACCCGTTTCAGAGTC‐3′ (product length 1090 bp).

### 4.16 FISH staining

In situ hybridization of bacterial rRNA on glass slides was performed as previously described (Becker et al., 2003) using the universal eubacterial oligonucleotide probe EUB‐338 (5′‐GCT GCC TCC CGT AGG AGT‐3′), containing a Cy3‐3 or FITC 5′‐modification (biomers). Slides with cryosections were fixed in PFA, washed in PBS, and incubated with 25 ng of each oligonucleotide added in 50 μl of hybridization buffer containing 20% formamide for 90 min at 46°C before washing with the same stringency. Signal specificity was demonstrated by using *Escherichia coli* as positive control with the EUB‐338 probe and by comparing with the non‐related Cy3‐labeled control NONEUB‐338 oligonucleotide and staining of gut samples derived from germ‐free animals.

### 4.17 Immunoblotting

Proteins were isolated from tissue and OMVs using Cell lysis buffer (Cell Signalling, Catalogue 9803) and from *E. coli* ^GFP/Cre^ culture (10 ml) using lysis buffer (50 mM Tris‐HCl pH7.5, 100 mM NaCl, 5 mM DTT) supplemented with 1 mM PMSF (Cell Signalling, Catalogue 8553). Bacterial lysates were prepared by additional sonification (10 times x 10 sec x 20 kHz on ice, Branson digital Sonifier). Lysates were centrifuged at 14,000 rpm for 20 min (4°C). Proteins were separated using a MiniProtean‐TGX gel (4‐15% polyacrylamide; Bio‐Rad) and transferred to a Nitrocellulose membrane (Bio‐Rad). Membranes were probed with the following primary antibodies against OmpC (Biorbyt, Orb6940, 1:1000 dilution). HRP‐linked anti‐rabbit (Cell Signalling, Catalogue 7074, 1: 10000 dilution) was used as a secondary antibody. For detection of OmpC, a 37.5 μl volume of purified OMV dissolved in PBS was mixed with 12.5 μl of 4X Laemmli buffer and 10% 2‐mercaptoethanol and boiled at 95C° for 5 min. The samples were then loaded into a MiniProtean‐TGX gel (4‐20% SDS‐PAGE; Bio‐Rad) and transferred onto a PVDF membrane (Bio‐Rad). The membrane was blocked with 5% non‐fat milk for 1 h and then incubated overnight in 4°C with anti‐OmpC in 1% milk. The membrane was then washed four times in PBS containing 0.1% Tween (PBST) and subsequently incubated with anti‐Rabbit IgG‐HRP for 1 h and washed four times in PBST. Signals were detected after incubation with Amersham ECL Prime substrate and imaged using an ImageQuant LAS 4000.

### 4.18 Liquid chromatography‐mass spectrometry (LC‐MS) analysis

Proteins were extracted using an SDS‐based buffer at a final concentration of 1.6% (w/v) SDS and digested using single‐pot solid‐phase‐enhanced sample preparation (SP3) as detailed before (Hughes et al., 2014; Sielaff et al., 2017). In brief, samples were first reduced and alkylated using dithiothreitol (DDT) and iodoacetamide (IAA), respectively. Excess IAA was quenched by the addition of DTT. Afterwards, 2 μl of carboxylate‐modified paramagnetic beads (Sera‐Mag SpeedBeads, GE Healthcare, 0.5 μg solids/ μl in water as described by Hughes et al. (Hughes et al., 2014) were added to the samples. After adding acetonitrile to a final concentration of 70 % (v/v), samples were allowed to settle at room temperature for 20 min. Subsequently, beads were washed twice with 70 % (v/v) ethanol in water and once with acetonitrile. Afterwards, beads were resuspended in 50 mM NH_4_HCO_3_ supplemented with trypsin (Mass Spectrometry Grade, Promega) and incubated overnight at 37°C. After overnight digestion, acetonitrile was added to the samples to reach a final concentration of 95 % (v/v). After 20 min of incubation at room temperature, beads were washed with acetonitrile. To recover bound peptides, paramagnetic beads were sonicated in 2 % (v/v) dimethyl sulfoxide (DMSO) in water for 1 min. After 5 min of centrifugation at 13,000 rpm (4 C), supernatants containing tryptic peptides were transferred into glass vials for MS analysis and acidified with 0.1 % (v/v) formic acid.

Tryptic peptides were analysed on an Ultimate 3000 RSLCnano LC system (Thermo Fisher Scientific) using a PEPMAP100 C18 5 μm 0.3 × 5 mm trap (Thermo Fisher Scientific) and an HSS‐T3 C18 1.8 μm, 75 μm x 250 mm analytical reversed‐phase column (Waters Corporation). Mobile phase A was water containing 0.1 % (v/v) formic acid and 3 % (v/v) DMSO. Peptides were separated running a gradient of 2–35 % mobile phase B (0.1 % (v/v) formic acid, 3 % (v/v) DMSO in ACN) over 90 min at a flow rate of 300 nl/min. Total analysis time was 120 min including wash and column re‐equilibration steps. Column temperature was set to 55°C. Mass spectrometric analysis of eluting peptides was conducted on an Orbitrap Exploris 480 instrument platform coupled to a FAIMS Pro interface (Thermo Fisher Scientific). Spray voltage was set to 2.2 kV, the funnel RF level to 40, and heated capillary temperature was at 275°C. Data were acquired in DDA mode using a fixed cycle time of 1.5s. Full MS resolution was set to 60,000 at *m/z* 200 and full MS automated gain control (AGC) target to 300 %. Mass range was set to *m/z* 350–1600. Fragment ion spectra were acquired with an AGC target value of 100 %. Resolution was set to 15,000 and ion transfer time was determined automatically (‘uto mode’). Normalized collision energy was fixed at 30 %. Data were acquired in positive mode. FAIMS voltages were set to ‐36 V and ‐48 V, respectively. Samples were analysed in triplicates in each FAIMS voltage.

Mass spectrometric data were processed and analysed using PEAKS Studio (version 10.6, Bioinformatics Solutions Inc.). Data were searched against a custom compiled *E. coli* proteome database (UniProtKB *E. coli* reference proteome release 2020) which contained a list of common contaminants and the sequence of the recombinase Cre (Bacteriophage P1). For database search, the following parameters were applied: (i) trypsin as enzyme for digestion, (ii) up to two missed cleavages per peptide, (iii) carbamidomethyl cysteine as fixed, and (iv) methionine oxidation as well as acetylation (N‐term) as variable modifications. The false discovery rate (FDR) for peptide and protein identification was assessed using the target‐decoy strategy by searching a reverse database and was set to 0.01 on both the protein and peptide level. Proteins had to be identified by at least two peptides.

All authors had access to the study data and reviewed and approved the final manuscript.

## AUTHOR CONTRIBUTIONS

M.B., S.M., R.L.R, and C.G. designed aspects of the research. M.B., P.R., S.L., I.S., A.D., S.M., S.U., T.A.P., M.N., N.K.V., S.T., U.D., I.S., V.R., R.L.R. performed the experiments. S.W., M.F.N. supplied material that made this study possible. M.B., R.L.R., S.M. and C.G. analyzed the data and wrote the paper.

## CONFLICT OF INTEREST

Authors declare no conflict of interest.

## Supporting information

Supplementary Figure 1. OMV Mass Spectrometry Analysis. (a) Venn diagram depicting overlap between most abundant proteins detected in OMV preparations (blue circle) and in UC supernatant (yellow circle). (b) Pie chart showing share of protein classes with highest abundance. (c) STRING network of proteins of *E. coli* ^Cre^ OMVs. The most abundant clusters are indicated by coloured circles.
Supplementary Figure 2. tdTomato positive cells in the intestinal epithelium. (a) Western blot analysis of iodixanol density gradient purified OMVs from *E. coli* ^Cre^ and *E. coli* ^GFP^. The membrane was probed with an antibody against OmpC (37 kDa). (b) Analysis of faecal CFUs of mice plated on LB and LB+Ampicillin Agar plates at day 0, 6 and 12 during the experiment. (c) Representative Maestro *ex vivo* images of the gastrointestinal tract of *Rosa26.tdTomato* mice treated with *E. coli* ^Cre^ or *E. coli* ^GFP^ as control analysed via multi‐spectral separation. Scale bar: 50 mm. (d) Detailed information on the calculation of data derived from volumetric reconstruction images of *Rosa26.tdTomato* intestinal mucosa treated with *E. coli* ^Cre^ (see Figure 3 (d+e)). (e) Graphical illustration of intestinal organoid generation from in vivo set up. (f) Representative immunohistochemical images of small intestinal (Ileum) cryo‐cross sections of *Rosa26.tdTomato* mice treated with *E. coli* ^Cre^. Confocal pictures visualized tdTomato‐positive cells (red) and staining with UEA‐1 (for Goblet cells) and E‐cadherin (CDH1 for epithelial cells). Nuclei counterstaining with Hoechst (blue). Scale bar: 100 μm. (g) Representative confocal z‐stack images of small intestinal organoids derived from *Rosa26.tdTomato* mice colonized with *E. coli* ^Cre^ (right) versus control (left) 2h post isolation. Scale Bar: 250 μm.
Supplementary Figure 3. Visualization of OMVs via OmpC in intestinal tissue. (a) Representative confocal image of small intestinal (Ileum) cryo‐cross section derived from a germ‐free animal stained with an antibody against OmpC to visualize OMVs and Phalloidin to stain actin filaments. Nuclei counterstaining with Hoechst (blue). Scale bar: 250 μm. (b) Representative pictures demonstrating OmpC staining in small intestinal cross sections derived from *Rosa26.tdTomato* mice that received *E. coli* ^Cre^ or were left unchallenged. Nuclei counterstaining with Hoechst (blue). Scale bar: 100 μm. (c) Representative confocal images of small intestinal (Ileum) cryo‐cross sections derived from *Rosa26.tdTomato* mice that received *E. coli* ^Cre^ stained with an antibody against OmpC to visualize OMVs and Phalloidin to stain actin filaments. Nuclei counterstaining with Hoechst (blue). Scale bar: 250 μm. (d) Relative mRNA expression in BMDMs cultured for 8 h in the absence (mock) or presence of OMVs with 2*10^9^OMVs/ml. Gene expression levels are shown relative to *Gapdh*. ****P < 0.0001, ***P < 0.001
Supplementary Figure 4. Barrier function is maintained in LPS treated animals. (a) Representative pictures of tdTomato positive resident liver cells. Scale bar: 50 μM. Arrows point towards tdTomato‐positive cells. BECs: biliary epithelial cells; HECs: hepatic epithelial cells; PV: portal vein; HA: hepatic artery; BD: bile duct; BDL: bile duct lumen. (b) Representative images of Fluorescence in situ hybridization (FISH) with a universal eubacterial probe (red/green) in the small intestinal, colon, spleen, liver, brain, kidney, and heart of *Rosa26.tdtomato* mice inoculated with *E. coli* ^Cre^ [Upper Panel] or *E. coli* ^Cre^ + *E. coli* ^GFP^ [Lower Panel] and treated with LPS. Hoechst 33342 (blue) was used for counterstaining. Scale bar: small intestine, colon, spleen: 250 μm; liver, brain, kidney, heart: 50 μm.
Supplementary Figure 5. Uncropped blots/gels

Supporting information.

Supporting information.
