# Supplementary material for: Visualizing transfer of microbial biomolecules by outer membrane vesicles in microbe‐host‐communication in vivo
Source: J Extracell Vesicles. 2021 Oct 19;10(12):e12159. doi: 10.1002/jev2.12159 (PMC8524437; doi:10.1002/jev2.12159)
Supplement: Supplementary file 1 — Supplementary Figure 1. OMV Mass Spectrometry Analysis. (a) Venn diagram depicting overlap between most abundant proteins detected in OMV preparations (blue circle) and in UC supernatant (yellow circle). (b) Pie chart showing share of protein classes with highest abundance. (c) STRING network of proteins of E. coli Cre OMVs. The most abundant clusters are indicated by coloured circles. Supplementary Figure 2. tdTomato positive cells in the intestinal epithelium. (a) Western blot analysis of iodixanol density gradient purified OMVs from E. coli Cre and E. coli GFP. The membrane was probed with an antibody against OmpC (37 kDa). (b) Analysis of faecal CFUs of mice plated on LB and LB+Ampicillin Agar plates at day 0, 6 and 12 during the experiment. (c) Representative Maestro ex vivo images of the gastrointestinal tract of Rosa26.tdTomato mice treated with E. coli Cre or E. coli GFP as control analysed via multi‐spectral separation. Scale bar: 50 mm. (d) Detailed information on the calculation of data derived from volumetric reconstruction images of Rosa26.tdTomato intestinal mucosa treated with E. coli Cre (see Figure 3 (d+e)). (e) Graphical illustration of intestinal organoid generation from in vivo set up. (f) Representative immunohistochemical images of small intestinal (Ileum) cryo‐cross sections of Rosa26.tdTomato mice treated with E. coli Cre. Confocal pictures visualized tdTomato‐positive cells (red) and staining with UEA‐1 (for Goblet cells) and E‐cadherin (CDH1 for epithelial cells). Nuclei counterstaining with Hoechst (blue). Scale bar: 100 μm. (g) Representative confocal z‐stack images of small intestinal organoids derived from Rosa26.tdTomato mice colonized with E. coli Cre (right) versus control (left) 2h post isolation. Scale Bar: 250 μm. Supplementary Figure 3. Visualization of OMVs via OmpC in intestinal tissue. (a) Representative confocal image of small intestinal (Ileum) cryo‐cross section derived from a germ‐free animal stained with an antibody again [file JEV2-10-e12159-s002.pdf]

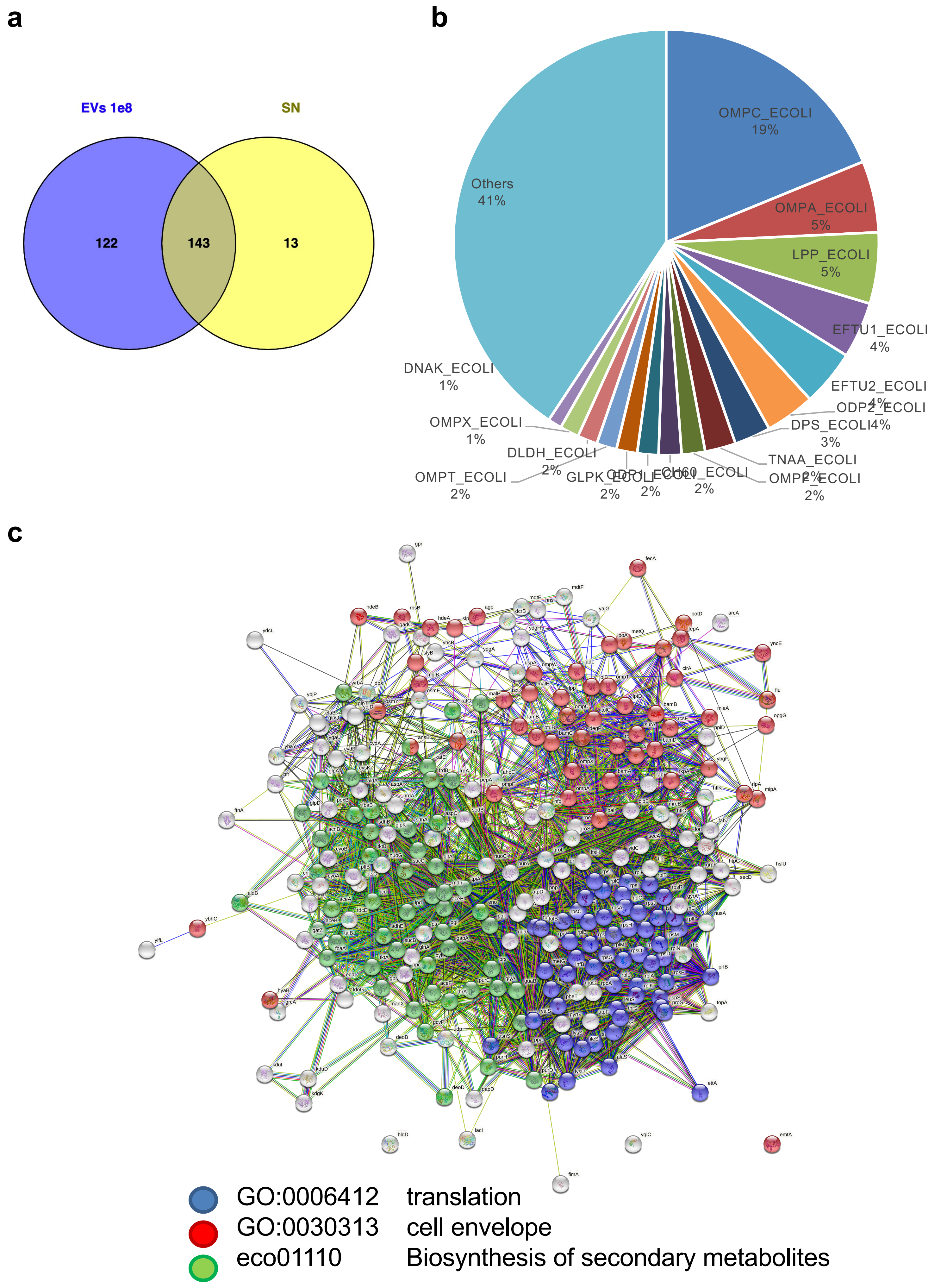

Supplementary Figure 1

a

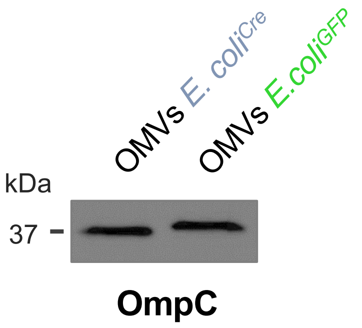

b

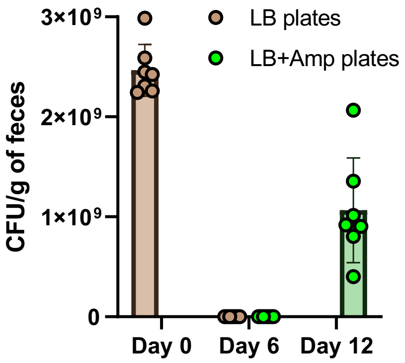

c

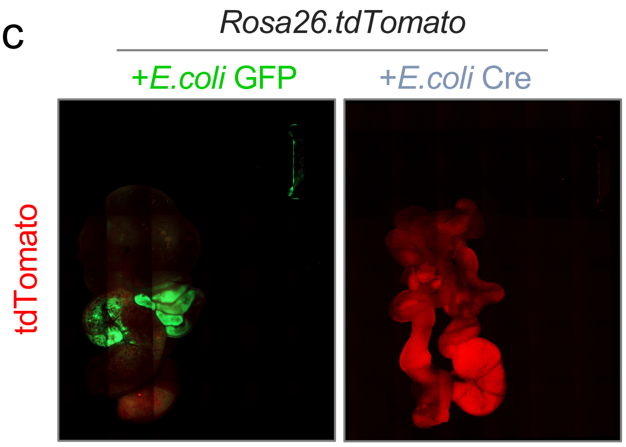

d

sample dimensions	sample area	total dimensions	total area	# crypts per sample area	# crypts per total area	# tdTom+ crypts per total area	% tdTom+ crypts per total area
0,43	0,157	2,14	6,66	78			
0,37		3,11		81			
				89			
			**MW:**	**83**	**3501**	**26**	**0,74**
0,43	0,157	2,09	6,75	70			
0,37		3,23		69			
				81			
			**MW:**	**73**	**3150**	**13**	**0,41**
0,43	0,157	2,34	7,04	66			
0,37		3,01		82			
				88			
			**MW:**	**78**	**3526**	**17**	**0,48**

e

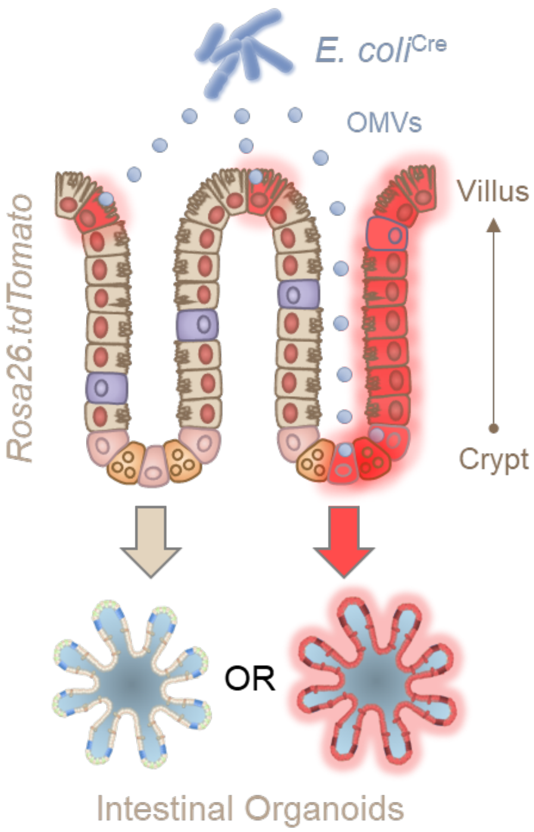

f

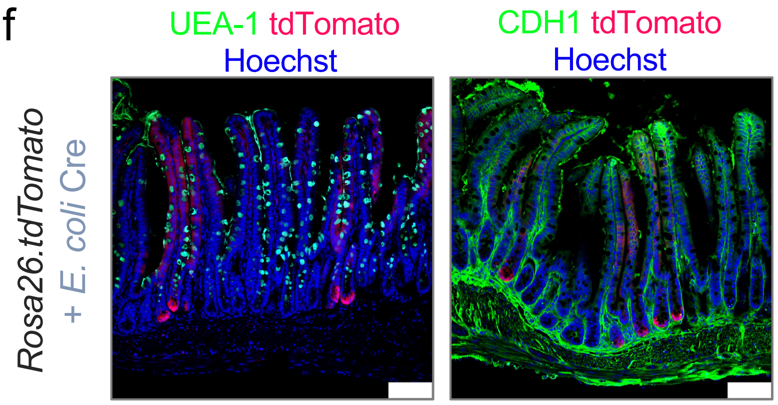

g

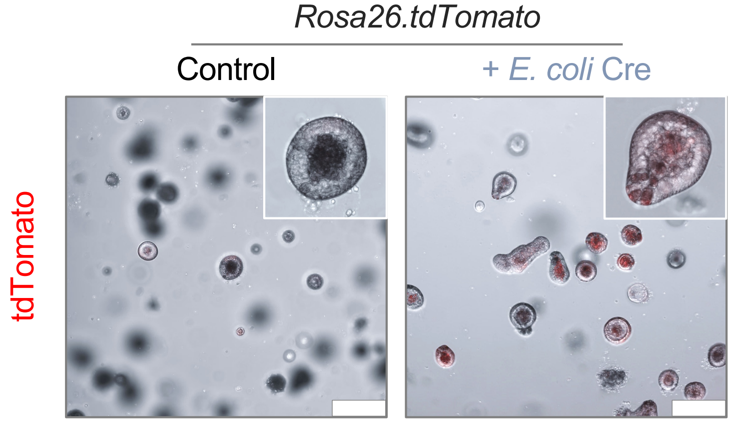

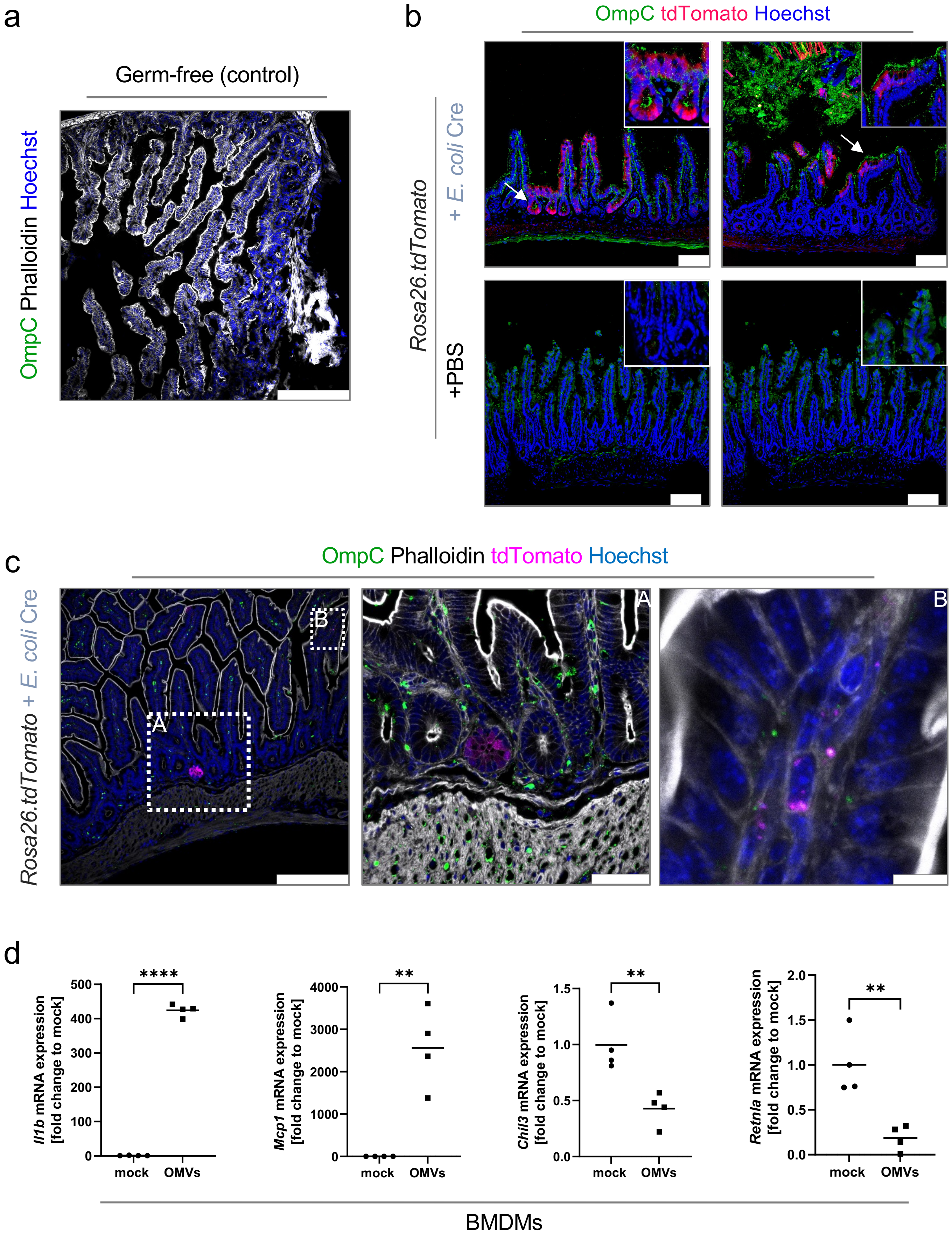

Supplementary Figure 3

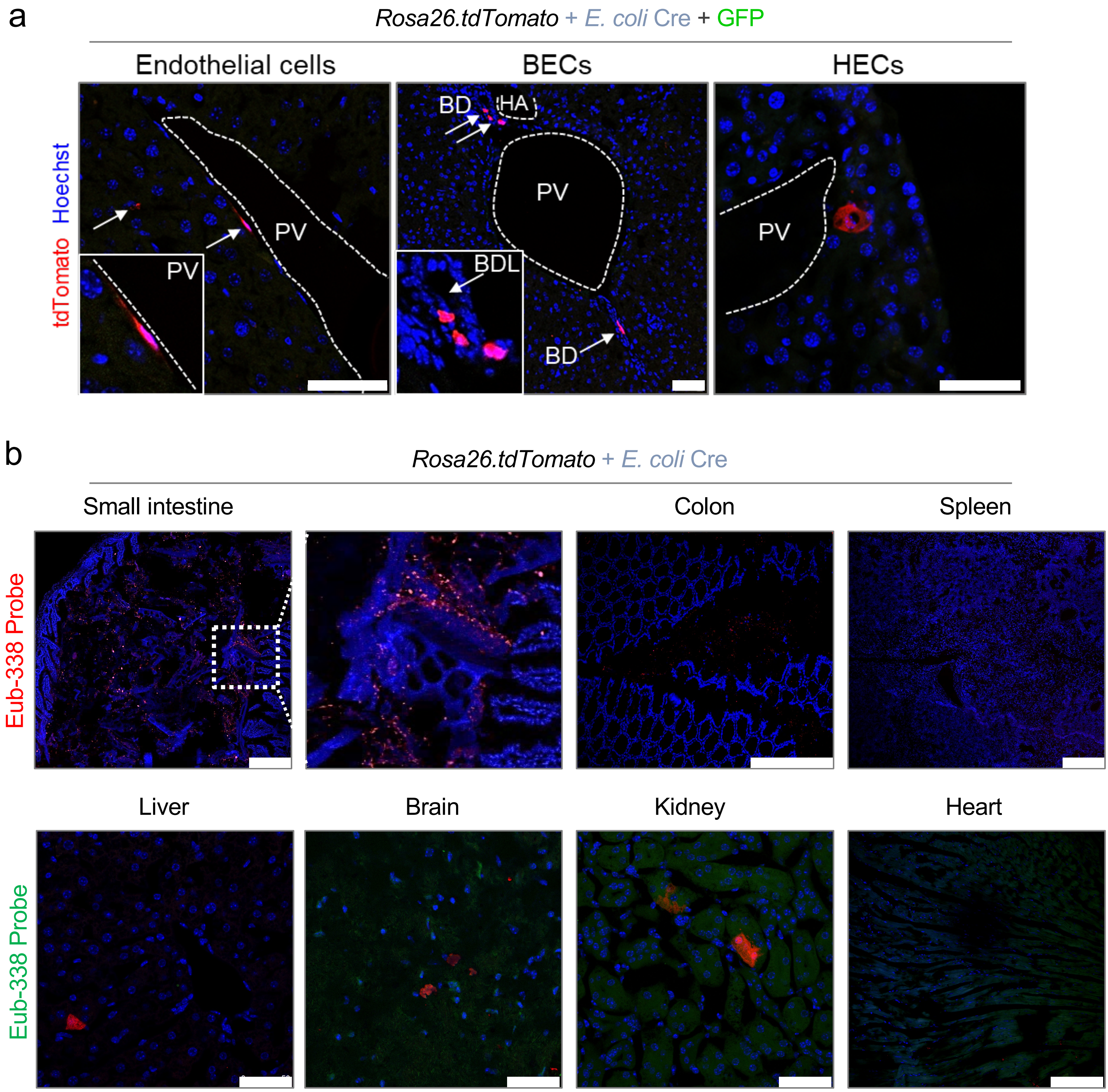
a
Rosa26.tdTomato + E. coli Cre + GFP
Endothelial cells
BECs
HECs
Hoechst
tdTomato
PV
PV
BD
HA
PV
BDL
BD
PV
b
Rosa26.tdTomato + E. coli Cre
Small intestine
Colon
Spleen
Eub-338 Probe
Liver
Brain
Kidney
Heart
Eub-338 Probe

Figure 1b (OmpC)

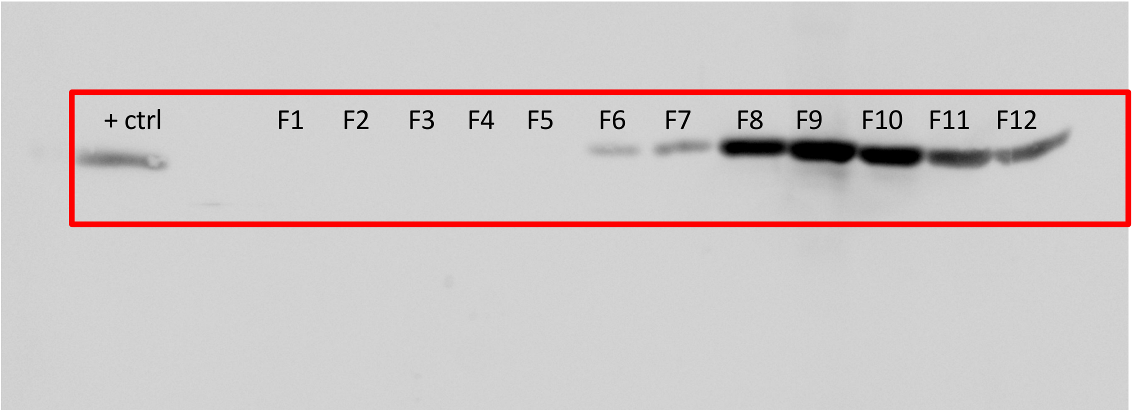

Supplementary Figure 2a (OmpC)

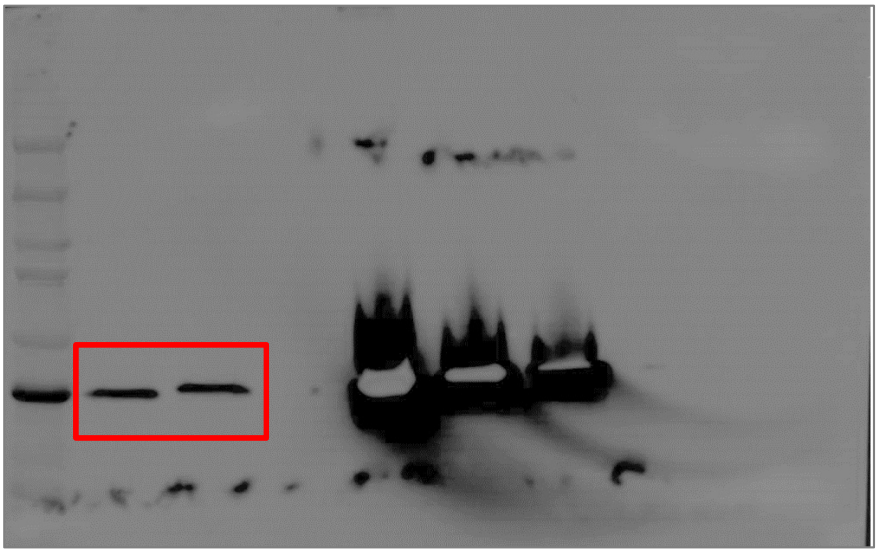

Supplementary Figure 5
